# GFPT1 deficiency in muscle leads to myasthenia and myopathy in mice

**DOI:** 10.1093/hmg/ddy225

**Published:** 2018-06-14

**Authors:** Yasmin Issop, Denisa Hathazi, Muzamil Majid Khan, Rüdiger Rudolf, Joachim Weis, Sally Spendiff, Clarke R Slater, Andreas Roos, Hanns Lochmüller

**Affiliations:** 1 John Walton Muscular Dystrophy Research Centre, MRC Centre for Neuromuscular Diseases, Institute of Genetic Medicine, Newcastle University, Newcastle, UK; 2 Leibniz-Institut für Analytische Wissenschaften—ISAS e.V, Dortmund, Germany; 3 Institute of Toxicology and Genetics, Karlsruhe Institute of Technology, Eggenstein-Leopoldshafen, Germany; 4 Interdisciplinary Center for Neurosciences, University of Heidelberg, Heidelberg, Germany; 5 Institute of Molecular and Cell Biology, Mannheim University of Applied Sciences, Mannheim, Germany; 6 Institute of Neuropathology, RWTH Aachen University Hospital, Aachen, Germany; 7 Institute of Neuroscience, Newcastle University, Newcastle, UK; 8 Department of Neuropediatrics and Muscle Disorders,Faculty of Medicine, Medical Center – University of Freiburg, Freiburg, Germany; 9 Centro Nacional de Análisis Genómico (CNAG-CRG), Center for Genomic Regulation, Barcelona Institute of Science and Technology (BIST), Barcelona, Catalonia, Spain

## Abstract

Glutamine-fructose-6-phosphate transaminase 1 (GFPT1) is the rate-limiting enzyme in the hexosamine biosynthetic pathway which yields precursors required for protein and lipid glycosylation. Mutations in *GFPT1* and other genes downstream of this pathway cause congenital myasthenic syndrome (CMS) characterized by fatigable muscle weakness owing to impaired neurotransmission. The precise pathomechanisms at the neuromuscular junction (NMJ) owing to a deficiency in GFPT1 is yet to be discovered. One of the challenges we face is the viability of *Gfpt1*^−/−^ knockout mice. In this study, we use Cre/LoxP technology to generate a muscle-specific GFPT1 knockout mouse model, *Gfpt1^tm1d^*^/^^*tm1d*^, characteristic of the human CMS phenotype. Our data suggest a critical role for muscle derived GFPT1 in the development of the NMJ, neurotransmission, skeletal muscle integrity and highlight that a deficiency in skeletal muscle alone is sufficient to cause morphological postsynaptic NMJ changes that are accompanied by presynaptic alterations despite the conservation of neuronal GFPT1 expression. In addition to the conventional morphological NMJ changes and fatigable muscle weakness, *Gfpt1^tm1d^*^/^^*tm1d*^ mice display a progressive myopathic phenotype with the presence of tubular aggregates in muscle, characteristic of the *GFPT1*-CMS phenotype. We further identify an upregulation of skeletal muscle proteins glypican-1, farnesyltransferase/geranylgeranyltransferase type-1 subunit α and muscle-specific kinase, which are known to be involved in the differentiation and maintenance of the NMJ. The *Gfpt1^tm1d/tm1d^* model allows for further investigation of pathophysiological consequences on genes and pathways downstream of GFPT1 likely to involve misglycosylation or hypoglycosylation of NMJs and muscle targets.

## Introduction

Congenital myasthenic syndromes (CMS) are a heterogeneous group of inherited disorders of neurotransmission that are commonly categorized according to the pathophysiological involvement in the presynaptic compartment, synaptic cleft and the postsynaptic basal lamina of the neuromuscular junction (NMJ). More recently, mutations in genes encoding ubiquitously expressed enzymes involved in glycosylation have also been implicated in CMS (5 out of 29 CMS causing genes) (1–9). 

Recessive mutations have been identified in the *GFPT1* gene that encodes glutamine-fructose-6-phosphate transaminase 1 (GFPT1) (7). GFPT1 is a ubiquitous protein that catalyses the conversion of fructose-6-phosphate to glucosamine-6-phosphate and glutamate via a transamidase reaction. This is the rate-limiting step in the hexosamine biosynthetic pathway which yields precursors required for N- and O-linked glycosylation of proteins (10). Splicing of *GFPT1* gives rise to two variants, a ubiquitous GFPT1 isoform and a long muscle-specific isoform, GFPT1-L, expressed predominantly in skeletal muscle and the heart (11). Missense mutations in *GFPT1* have been found outside of the muscle-specific exon, yet impaired function seems to be restricted to the muscle and NMJ. 

Patients with mutations in *GFPT1* demonstrate a progressive limb-girdle pattern of weakness, tubular aggregates in skeletal muscle, an unusual sparing of the ocular and bulbar muscles, and an improvement in symptoms in response to cholinesterase inhibitors (2,7,8). Some patients also display a decremental response to electromyography (EMG) (2,12) and repetitive nerve stimulation (7–9), and endplates in patient biopsies display simplification of the postsynaptic membrane with fewer and poorly developed junctional folds (2,8,9,12). Until now there has only been one report of a human mutation that disrupts the GFPT1-L isoform resulting in the absence of glycosylated proteins. A patient biopsy of the anconeus muscle showed abnormal variation of fibre size, sparse regenerating and necrotic fibres, vacuolated fibres, endomysial fibrosis and densely packed membranous tubular aggregates (2). 

The precise mechanism whereby the NMJ is affected by defects in glycosylation is not clear. One hypothesis is that mutations in glycosylation enzymes impair the glycosylation of proteins involved in the formation and maintenance of the NMJ and neurotransmission. Here, we report the first CMS mouse model depicting defective glycosylation. By generating a muscle-specific GFPT1 deficient mouse model we established a good phenocopy of the human disorder enabling deeper functional and biochemical studies of the underlying pathology which leads to the *GFPT1*-CMS phenotype.

## Results

### Generation of the GFPT1 muscle-specific knockout mouse

**Figure 1.**
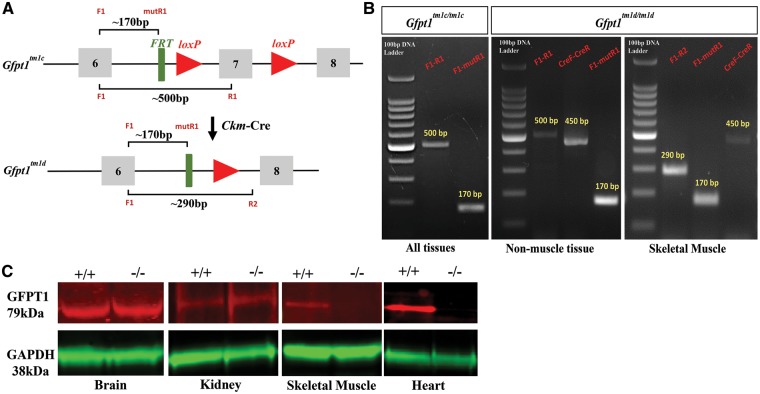
Generation of muscle-specific *Gfpt1* knockout mice. (**A**) Schematic demonstrating the genomic region of the *Gfpt1* gene where Exon 7 is floxed by loxP sites. Conversion of the *Gfpt1^tm1c^* allele to the *Gfpt1^tm1d^* allele in skeletal and cardiac muscle of *Gfpt1* mutant mice carrying a *Cre* transgene under the control of the *Ckm* promoter. (**B**) PCR demonstrating recombination events that take place in muscle and non-muscle tissues of *Gfpt1^tm1d^*^/^^*tm1d*^ mice. (**C**) Protein lysates from brain, kidney, skeletal muscles and the heart of control and mutant mice were analysed by western blotting against a GFPT1 antibody. An antibody against GAPDH was used as a loading control (*n* = 6).

The basic targeting strategy used in this study has been described previously (13). The initial breeding and genotyping steps required for this study were carried out in-house (Supplementary Material, Resources S1–S3). To generate a conditional *Gfpt1* knockout allele, homozygous mice harbouring two loxP sites flanking exon 7 of the *Gfpt1* gene (*Gfpt1^tm1c/tm1c^*) were bred with mice harbouring the *Ckm-Cre* transgene [B6.FVB(129S4)-Tg(Ckmm-cre)5Khn/J]. These mice express Cre recombinase under the control of the *Ckm* promoter (14). Homozygous mice with this genotype will be referred to as *Gfpt1^tm1d/tm1d^*. Cre-mediated recombination results in the deletion of *Gfpt1* in skeletal and cardiac muscle only (Fig. 1A). To confirm the spatial and temporal expression of Cre recombinase we used the *lacZ* reporter mouse line ROSA26R-*lacZ* that demonstrates the activity of the *Ckm* promoter. Our findings confirmed *Ckm*-Cre expression in skeletal and cardiac muscle only (data not shown). Mice homozygous for *Gfpt1^tm1c/tm1c^* were used as controls. Cre-mediated gene alteration in muscle of *Gfpt1* mutant mice was confirmed by genomic polymerase chain reaction (PCR). Primers were designed to detect the deletion of Exon 7. PCR on genomic DNA extracted from muscle and non-muscle tissues from *Gfpt1^tm1c^*^/^^*tm1c*^ mice produce fragments of ∼500 bp. DNA amplified from *Gfpt1^tm1d^*^/^^*tm1d*^ mice produce fragments of ∼500 bp in non-muscle tissues and a truncated band of ∼290 bp in skeletal and cardiac muscle in the presence of *Cre* (∼450 bp). All targeted alleles produce fragments of ∼170 bp (Fig. 1B). The expression of GFPT1 in tissues was examined by western blotting using a polyclonal antibody against GFPT1. GFPT1 (∼79 kDa) is expressed in muscle and non-muscle tissues in control mice. Results confirmed the absence of GFPT1 in skeletal and cardiac muscle from *Gfpt1^tm1d^*^/^^*tm1d*^ mice, whilst maintaining expression in the brain and kidney. Glyceraldehyde 3-phosphate dehydrogenase (GAPDH) (∼38 kDa) was used as a loading control (Fig. 1C).

### *Gfpt1^tm1d/tm1d^* mice demonstrate signs of muscle weakness and fatigue

**Figure 2.**
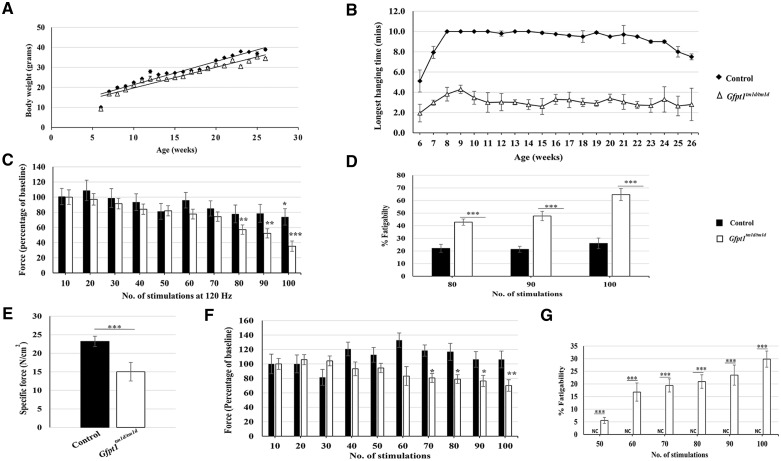
A comparison of body weight and muscle strength between control and *Gfpt1^tm1d/tm1d^* mice. (**A**) Growth curves demonstrating changes in body weight over a 6 months period (*n* = 8). (**B**) Quantitative analysis of latency to fall from a wire grid at various time points up to the age of 6 months (*n* = 8). *Gfpt1^tm1d/tm1d^*mice perform worse than control mice at all time points (*P* < 0.01). (**C**) Quantitative analysis of force generated by the TA muscle after every 10 stimulations of the CPN in 3 months old control and *Gfpt1^tm1d/tm1d^* mice (*n* = 5). Data are expressed as a percentage of baseline force. (**D**) Quantification of fatigability of the TA muscle after 80, 90 and 100 stimulations of the CPN in 3 months old control and *Gfpt1^tm1d/tm1d^*mice (*n* = 5). Data are expressed as the percentage decrease of baseline force. (**E**) Isometric tetanic maximal force in diaphragm muscle from control and *Gfpt1^tm1d/tm1d^* mice (*n* = 4). (**F**) Quantitative analysis of force generated by the diaphragm muscle after every 10 stimulations in 3 months old control and *Gfpt1^tm1d/tm1d^* mice (*n* = 4). Data are expressed as a percentage of baseline force. (**G**) Quantification of the fatigability of the diaphragm muscle between 50 and 100 stimulations in 3 months old control and *Gfpt1^tm1d/tm1d^*mice. Data are expressed as a percentage reduction in force. Data are mean±SEM. NC, no change; *P < 0.05, **P < 0.01, ***P < 0.001.

*Gfpt1^tm1d/tm1d^* mice do not exhibit gross phenotypical defects when compared with age-matched control mice. Although the general trend shows that *Gfpt1^tm1d/tm1d^* mice are slightly smaller when compared with control mice, no significant differences in body weight were observed between control and *Gfpt1^tm1d/tm1d^* mice over the course of development (Fig. 2A).

To assess any changes in motor function we performed the inverted screen test assay over a period of 6 months. Control mice up to the age of 18 weeks demonstrate the ability to hold on for the maximum set threshold of 10 min. There was a small reduction in the latency to fall in older mice that can be accounted for by an increase in body weight. *Gfpt1^tm1d/tm1d^* mice demonstrate poor motor performance as early as 6 weeks old onwards, shown by a reduction in the latency to fall from the grid compared with controls (latency decrease: 8 weeks old, 62%; 12 weeks old, 69%; 16 weeks old, 66%; 20 weeks old, 64%; 24 weeks old, 63%; Fig. 2B). The deficit in motor performance is not progressive over time. We further demonstrate there is no change in body weight and hang time between control mice (Cre negative) and mice carrying the Cre transgene (Supplementary Material, Resource S4). To confirm the presence of muscle weakness in *Gfpt1^tm1d/tm1d^* mice, we studied the ability of tibialis anterior (TA) muscles from 3 months old control and *Gfpt1^tm1d/tm1d^* mice to evoke tetanic contractions in response to stimulation of the sciatic nerve *in situ*. Analysis of specific force [force normalized to cross-sectional area (CSA) of muscle] revealed no significant differences in muscle strength between control and *Gfpt1^tm1d/tm1d^*mouse muscle (Supplementary Material, Resource S5). We assessed muscle fatigue over a total of 100 tetanic nerve stimulations at 120 Hz (the frequency that resulted in *P*_o_). Muscle fatigue was observed in both control and *Gfpt1^tm1d/tm1d^*mice (Fig. 2C). After 100 stimulations, control mice demonstrated a 26.1% reduction in force produced compared with baseline, whereas *Gfpt1^tm1d/tm1d^*mice showed a 64.7% deficit, exhibiting a more pronounced degree of fatigue. A progressive decrease in force produced in *Gfpt1^tm1d/tm1d^*mice is evident from 80 stimulations (80, 42.9%; 90, 47.7%; 100, 64.7%), whereas a significant reduction of force in control mice is only evident following 100 stimulations compared with baseline. A comparison of fatigability between control and *Gfpt1^tm1d/tm1d^*mice showed that mutant mice exhibit a significant and progressive reduction in force produced after 80 (20.8%), 90 (26.4%) and 100 (38.7%) stimulations (Fig. 2D). Our data demonstrate that whilst deletion of GFPT1 does not have a pronounced effect on TA muscle strength, the muscle is more susceptible to fatigue.

### Contractile properties of *Gfpt1^tm1d/tm1d^* diaphragm muscles

We assessed the contractile properties of diaphragm muscles from *Gfpt1^tm1d/tm1d^*mice using an *in vitro* test apparatus. We examined the ability of muscle to contract following a train of tetanic stimulations. Both control and *Gfpt1^tm1d/tm1d^* muscles maintained tetanic contractions with stimulation frequencies ranging from 30 to 150 Hz. However, *Gfpt1^tm1d/tm1d^* muscles develop less force than control ones (Fig. 2E). Quantitative analysis revealed that the maximal isometric tetanic force was significantly reduced compared with controls (150 Hz, 35.3%). We tested for fatigue using a series of 100 tetanic nerve stimulations at 150 Hz (the frequency resulting in *P*_o_). We did not observe signs of fatigue in control diaphragms, but we see a progressive reduction in force between 50 and 100 stimulations in *Gfpt1^tm1d/tm1d^*diaphragms (Fig. 2F) (50, 5.6%; 60, 16.8%; 70 19.4%; 80, 20.9%; 90, 23.5%; 100, 29.8%). We compared the reduction in force produced between control and *Gfpt1^tm1d/tm1d^*mice expressed as percentage fatigability. Since we do not observe a deficit in force in control mice, *Gfpt1^tm1d/tm1d^*mice produced significantly less force from 50 to 100 stimulations compared with controls (50, 5.6%; 60, 16.8%; 70, 19.4%; 80, 20.9%; 90, 23.5%; 100, 29.8; Fig. 2G).

### Histological changes in skeletal muscle from *Gfpt1^tm1d/tm1d^* mice

**Figure 3.**
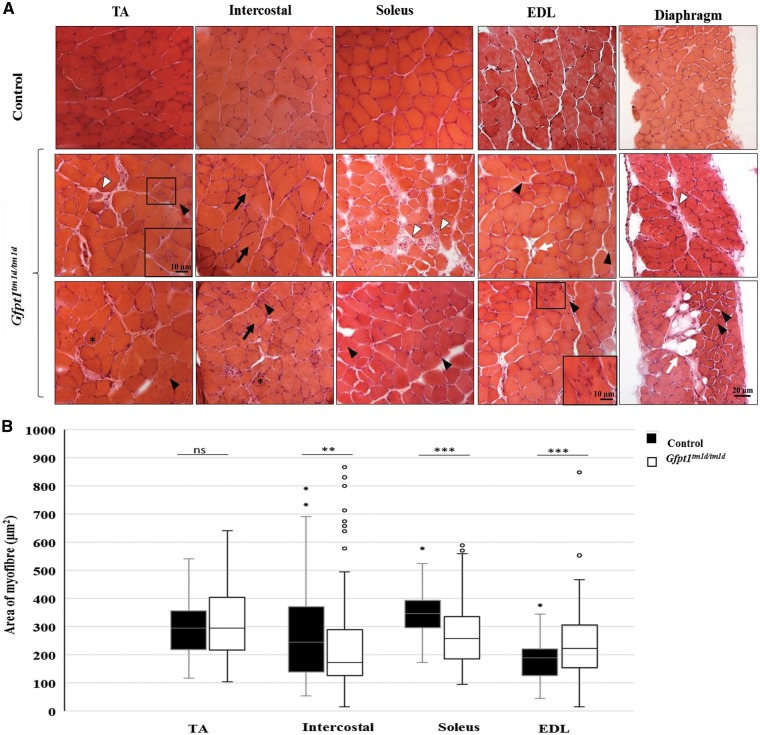
Characteristics and myopathic changes in muscle from *Gfpt1^tm1d/tm1d^* mice. (**A**) Representative TA, intercostal, soleus, EDL and diaphragm muscles stained with haematoxylin and eosin from control and *Gfpt1^tm1d/tm1d^* mice. Rounded myofibres (asterix), centrally located nuclei in myofibres (black arrows), tubular aggregates (black arrow head), necrotic fibres (white arrow head), adipose tissue (white arrows). Hypertrophic and atrophic myofibres are also present. (**B**) Quantitative analyses demonstrating the distribution of myofibre size according to CSA. Data are median, 25th percentile, 75th percentile, minimum and maximum values (including outliers). **P < 0.01; ***P < 0.001; ns, not significant (Mann–Whitney *U* test) (*n* = 4).

**Figure 4.**
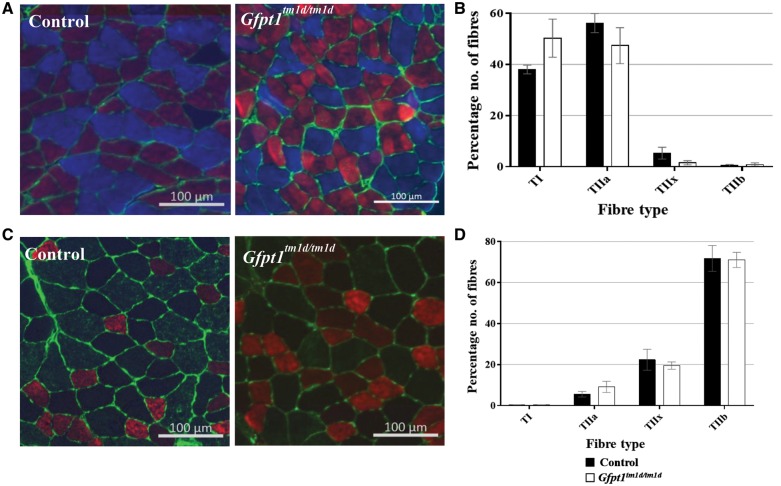
Fibre type labelling in control and *Gfpt1^tmld/tmld^* mouse muscle. Soleus (**A**) and TA (**C**) muscles were labelled with antibodies against MHC: Type 1 (blue) I, Type IIa (red), Type IIb (green) and laminin (green). Type IIx fibres were labelled on a second section (not shown). There were no differences in the fibre type proportion in both the soleus (*n* = 4) (**B**) and TA muscles (*n* = 4) (**D**).

Haematoxylin and eosin staining on TA, intercostal, soleus, extensor digitorum longus (EDL) and diaphragm muscles demonstrate myopathic changes in *Gfpt1^tm1d/tm1d^* mouse (Fig. 3A). Whilst control mouse muscle fibres maintain their characteristic polygonal shape and peripherally located nuclei, muscles from *Gfpt1^tm1d/tm1d^* mice exhibit occasional rounded myofibres (asterix), a small number of fibres with internal nuclei (black arrows), necrotic fibres (white arrow head) and the presence of atrophic and hypertrophic myofibres, some of which exhibit splitting. *Gfpt1^tm1d/tm1d^* mouse muscle also presented with tubular aggregates in some myofibres in all muscles examined (black arrow head), together with the replacement of myofibres with adipose tissue (white arrows), predominantly found in the diaphragm muscle. Quantitative analysis of the CSA of muscle fibres showed higher variability in *Gfpt1^tm1d/tm1d^* mouse muscle when compared with control mouse muscle (Fig. 3B). Differences in fibre size between control and *Gfpt1^tm1d/tm1d^* mouse muscle are greatest in the EDL (61%), followed by the soleus (56%), intercostal (29%) and TA (26%) muscle, as shown by the percentage difference of the interquartile range. Median CSA measurements are indicative of the proportion of fibres that tend to be either smaller or larger when comparing control and mutant muscles. The intercostal and soleus muscles exhibit a shift towards smaller fibres (29% and 26%, respectively), the EDL exhibits a shift towards larger fibres (18%) and the TA fibres remain unchanged in *Gfpt1^tm1d/tm1d^* mice. No differences were observed in the proportion of fibre types in the soleus (Fig. 4A and B) or TA (Fig. 4C and D) muscles between control and mutant mice. Further analyses revealed no significant tubular aggregate fibre-type-specific predominance in the soleus and TA muscles (Supplementary Material, Resource S6). No pathological changes were observed in cardiac muscle from *Gfpt1^tm1d/tm1d^*mice compared with controls (data not shown).

### *Gfpt1^tm1d^* ^/^^*tm1d*^ mice exhibit presynaptic and postsynaptic remodelling

**Figure 5.**
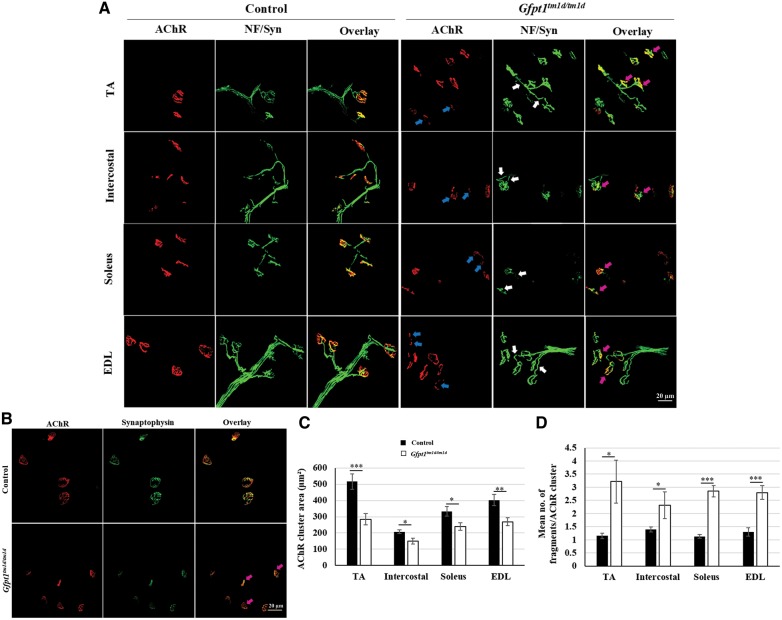
Aberrant NMJs in 3 months old *Gfpt1^tm1d^*^/^^*tm1d*^ mice. (**A**) Whole-mount TA, intercostal, soleus and EDL muscles were labelled with anti-neurofilament, anti-synaptophysin and Alexa fluor 594 α-bungarotoxin. (**B**) Whole-mount TA muscles labelled with anti-synaptophysin and Alexa fluor 594 α-bungarotoxin demonstrating the degree of co-localization of pre- and postsynaptic components. Quantitative analysis demonstrating AChR cluster area (**C**), and mean number of fragments/AChR cluster (**D**) in control and *Gfpt1^tm1d^*^/^^*tm1d*^ mice (*n* = 6). AChR fragments (blue arrows), discontinuous and disorganized axons (white arrow), presynaptic and postsynaptic overlap (magenta arrows). Data are mean±SEM. *P < 0.05, **P < 0.01, ***P < 0.001; ns, not significant.

We analysed the pre- and post-synaptic molecular structure of NMJs in TA, intercostal, soleus and EDL muscles from 3 months old mice. Whole-mount muscles were stained with α-bungarotoxin to label acetylcholine receptor (AChR) clusters, and with antibodies against neurofilament and synaptophysin to label axonal branches and nerve terminals, respectively. In control mice, axonal branches project normally and innervate the well-defined ‘pretzel’-like AChR cluster. AChR clusters from *Gfpt1^tm1d/tm1d^* mouse muscle do not maintain this characteristic shape, but instead appear smaller and fragmented when compared with AChRs from control mice (blue arrows). *Gfpt1^tm1d/tm1d^* mice also exhibit presynaptic changes including the appearance of discontinuous and disorganized axonal projections (white arrows). Nevertheless, axons can project normally to endplates and do not show any signs of overshooting, retractions or axonal sprouting (magenta arrows) (Fig. 5A). Synaptophysin labelling shows fragmented nerve terminals in *Gfpt1^tm1d^*^/^^*tm1d*^ mice that align with existing AChR cluster fragments to form synaptic contacts (magenta arrows) (Fig. 5B). Quantitative analysis revealed a reduction in AChR cluster area, which was greatest in the TA (45%), followed by the EDL (33%), soleus (28%) and intercostal (27%) muscles (Fig. 5C). The mean number of AChR fragments was greater in muscles from *Gfpt1^tm1d/tm1d^* mice than in controls (control versus *Gfpt1^tm1d/tm1d^*; TA: 1.2 versus 3.2, intercostal: 1.4 versus 2.3, soleus 1.1 versus 2.9, EDL: 1.3 versus 2.8) (Fig. 5D). Statistical analysis revealed the greatest degree of fragmentation in the soleus, followed by the EDL, intercostal and TA muscles. The nerve terminals also exhibit some degree of remodelling in comparison to controls.

**Figure 6.**
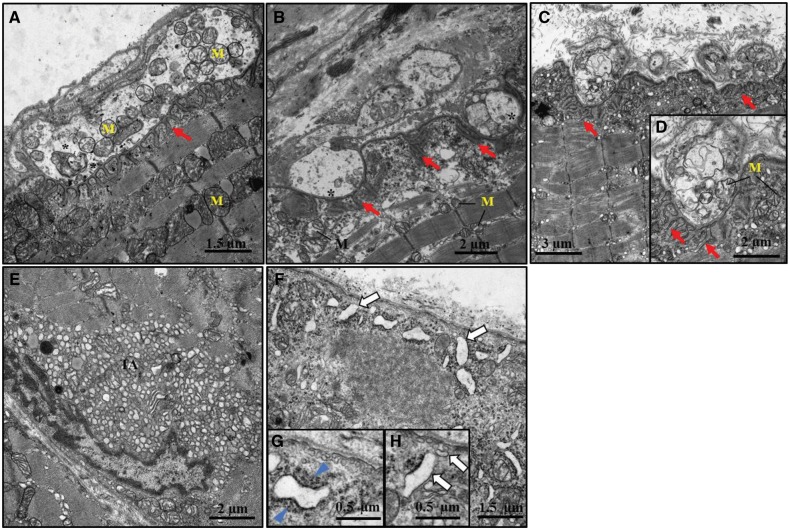
Altered morphology at the ultrastructural level in *Gfpt1^tm1d^*^/^^*tm1d*^ mouse muscle. Representative electron micrographs from 3 months old control and *Gfpt1^tm1d^*^/^^*tm1d*^ intercostal muscles. Examples of control (**A**), and *Gfpt1^tm1d^*^/^*^tm1d^*(**B**–**D**) NMJs. Tubular aggregates (**E**) and subsarcolemmal vesicular structures (**F**–**H**) in *Gfpt1^tm1d/tm1d^* mouse muscle. Synaptic vesicles (*), junctional folds (red arrow), mitochondria (M), tubular aggregates (TA), subsarcolemmal vesicular structures (white arrow), dense filamentous-like material (blue arrowhead) (*n* = 4).

To gain an enhanced understanding of the NMJ phenotype, we used electron microscopy to examine endplates at the ultrastructural level in intercostal muscles from 3 months old control (Fig. 6A) and mutant mice (Fig. 6B–H). NMJs in *Gfpt1^tm1d/tm1d^* mice displayed fewer numbers of junctional folds per terminal (red arrows) (control mean ±SEM: 13.14 ± 1.23 versus *Gfpt1^tm1d/tm1d^*: 8.71 ± 1.70) (Fig. 6B–D;Supplementary Material, Resource S7) on the postsynaptic membrane and an abundant accumulation of tubular aggregates beneath the sarcolemma in mutant mice (Fig. 6E). We also see the presence of accumulated subsarcolemmal vesicular structures that resemble enlarged caveolae with impaired sarcolemmal fusion (white arrows) (Fig. 6F–H), and proliferated and/or widened sarcoplasmic reticulum and Golgi structures as well as deposits of dense filamentous-like material (blue arrowhead), most likely corresponding to aggregated protein (Fig. 6G and H). In small pre-terminal intramuscular nerve fascicles from *Gfpt1^tm1d/tm1d^*mice, we observed highly irregular convoluted myelin sheaths surrounding some axons, associated with a mean reduction in the diameter of myelin sheaths when compared with controls (control: 0.52 ± 0.03 µm; *Gfpt1^tm1d/tm1d^*: 0.32 ± 0.01 µm) (Supplementary Material, Resource S7). Further analysis revealed that the number of mitochondria per nerve terminal remains unaffected (data not shown). Collectively, our data indicate the presence of both pre- and postsynaptic alterations of the NMJ and muscle in *Gfpt1^tm1d/tm1d^* mice.

### Effects of GFPT1 deficiency on caveolin-3

**Figure 7.**
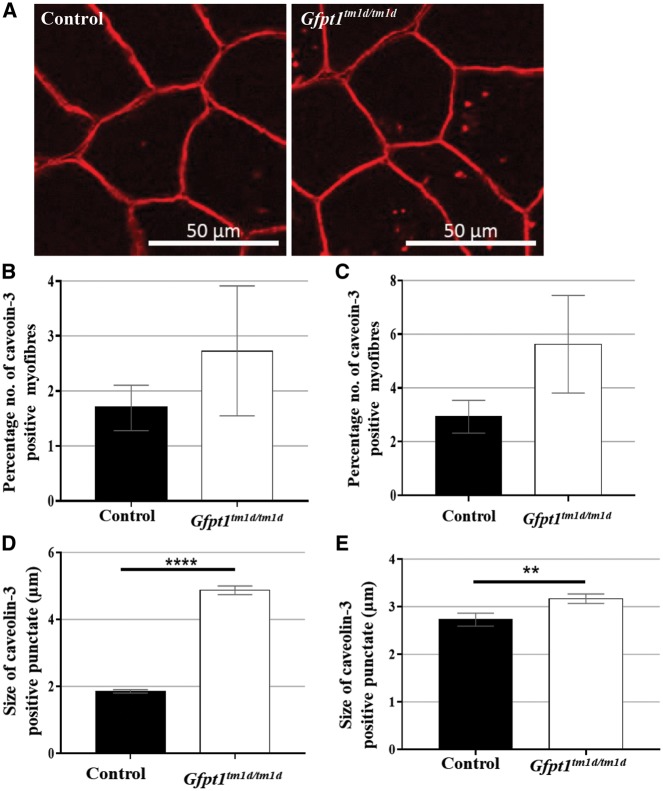
Caveolin-3 expression in muscles from control (*n* = 3) and *Gfpt1^tm1d/tm1d^* (*n* = 4) mice. Transverse sections of the soleus (**A**) and TA muscles were labelled with an antibody against caveolin-3. No significant differences in the percentage of myofibres expressing caveolin-3 were observed in the soleus (**B**) and TA (**C**) muscles from *Gfpt1^tm1d/tm1d^* mice compared with controls. Caveolin-3 positive punctate were significantly larger in *Gfpt1^tm1d/tm1d^* soleus (**D**) and TA (**E**) muscles compared with controls. Data are mean±SEM. **P < 0.01, ****P < 0.0001.

Our electron microscopy data showing the presence of ectopic subsarcolemmal caveolae-like vesicular structures indicative of ER-Golgi-stress prompted further investigation into the subcellular distribution and prevalence of caveolin-3 in muscles. Non-sarcolemmal caveolin-3 positive puncta were observed in both control and *Gfpt1^tm1d/tm1d^* mouse muscle (Fig. 7A), with no significant differences in the percentage of myofibres showing caveolin-3 immunoreactivity in the soleus and TA muscles from *Gfpt1^tm1d/tm1d^* mice compared with controls (Fig. 7B and C). Quantitative analysis of immunofluorescent cytoplasmic punctate staining corresponding to caveolin-3 revealed significantly larger puncta in *Gfpt1^tm1d/tm1d^* soleus and TA muscles compared with controls (Fig. 7D and E).

### Stability of AChR remains unaffected in *Gfpt1^tm1d^*^/^^*tm1d*^ mice

To establish whether the stability of AChRs is compromised in *Gfpt1^tm1d/tm1d^* mice, we assessed the turnover rate of AChRs in the TA muscle of 3 months old control and mutant mice over a 10-day period. To avoid muscle paralysis, a non-saturating 25 pmol dose of α-bungarotoxin was used for labelling ‘old’ and ‘new’ receptors labelled green and red, respectively. Fluorescence signals were monitored using confocal microscopy. Quantitative assessment of relative pixel intensities using automated image analysis demonstrates no significant difference between *Gfpt1^tm1d/tm1d^* and control mice (Supplementary Material, Resource S8).

### Effects of GFPT1 deficiency on the intercostal muscle proteome

**Figure 8.**
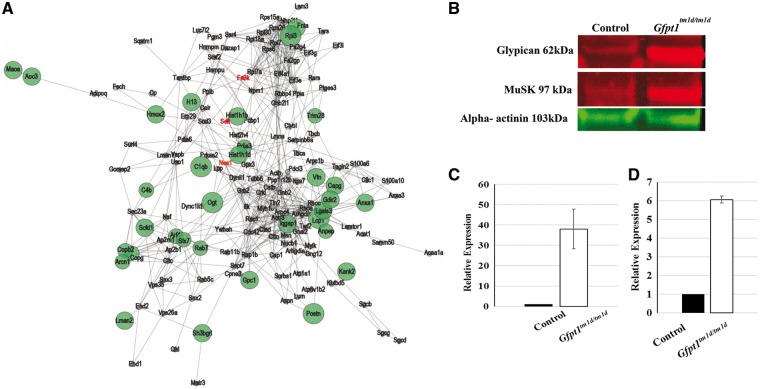
Proteins regulated as a consequence of GFPT1 depletion in 3 months old intercostal muscles. (**A**) STRING analyses demonstrating the spectrum of affected proteins. Proteins that show upregulation (green) and downregulation (red) are shown. (**B**) Immunoblot studies show upregulation of glypican-1 and MuSK proteins in *Gfpt1^tm1d/tm1d^* mice. Quantitative analysis showing the relative expression levels of glypican-1 (**C**) (*n* = 3), and MuSK (**D**) (*n* = 2) normalized to their corresponding α-actinin loading controls.

Proteomics is a powerful tool for the unbiased investigation of pathophysiological processes in neuromuscular disorders (15). Here, we compared intercostal muscles from 3 months old control and *Gfpt1^tm1d/tm1d^* mice using quantitative mass spectrometry (label-free proteomic approach). We found that 2.8% of the quantified proteins (43 out of 1517) were differentially expressed upon GFPT1 deficiency in intercostal muscles. Thirty-nine of these proteins were upregulated (29 identified with a minimum of 2 unique peptides and 10 with just 1 unique peptide) and 4 downregulated (all identified with 1 unique peptide). Five out of the 39 upregulated proteins and 1 out of the 4 downregulated proteins harbour *N*-glycosylation (*N*-GlcNAc) sites. One out of the four downregulated proteins harbour *O*-glycosylation (*O*-GlcNAc) sites (Supplementary Material, Resource S9). Most of the affected proteins are localized in the ER-Golgi network, plasma membrane, cytoplasm, nucleus and mitochondria (Supplementary Material, Resources S10 and S11). An overview of the regulated proteins and proposed functions is given in (Supplementary Material, Resource S12). Proteins of interest include glypican-1, muscle-specific kinase (MuSK) and farnesyltransferase/geranylgeranyltransferase type-1 subunit α (FNTA). To provide insight into GFPT1 myopathology, the spectrum of affected proteins was analysed for enriched gene ontology terms using STRING (Fig. 8A), and a pathway analysis was performed based on functions described in uniprot (16) and ‘Reactome’ (Supplementary Material, Resource S13). We have also verified the proteomic findings for glypican-1 and identified increased protein abundance of MuSK in the muscles derived from *Gfpt1* mutant animals using immunoblot studies (Fig. 8B–D).

## Discussion

Owing to the ubiquitous nature of GFPT1 and its expression in the early stages of embryonic development, we confirmed that complete knockout of this protein results in embryonic lethality as homozygous GFPT1 knockout mice were never obtained through breeding heterozygous knockout mice. In this study, we report the generation of a Cre/loxP-mediated conditional GFPT1 knockout mouse model and characterize the adult phenotype resulting from a deficiency of GFPT1 in skeletal muscle only. Prior to this study it was unknown whether depletion of GFPT1 in muscle only would be sufficient to produce a CMS-like phenotype. Most pathogenic variants identified in patients with *GFPT1*-CMS affect both GFPT1 isoforms resulting in reduced expression of the GFPT1 protein (10). To date, there has only been one report of a patient harbouring a nonsense mutation in the ubiquitous GFPT1 isoform and a second splice-site mutation causing a frameshift at the muscle-specific GFPT1 exon, leading to a complete loss of the muscle-specific isoform only (2).

The strategy used to generate the mouse model described in this study was designed to disrupt both GFPT1 isoforms in muscle only. Full realization of our model relies on our understanding of a temporally regulated series of events occurring in skeletal muscle of the developing mouse embryo. Formation of the NMJ begins with pre-patterning of AChRs from embryonic day 12.5, and by embryonic day 18.5 AChR clusters have differentiated and are well innervated (17,18). The expression of muscle creatine kinase has been reported in skeletal and cardiac muscle of the mouse embryo 13 days post coitum, which rapidly increases by embryonic day 15 (19). The expression of *Ckm*-Cre, and hence the depletion of GFPT1 temporally correlates with events taking place during the formation of the NMJ. Consequently, any phenotypes we observe in the GFPT1 mouse model reflects the pathophysiology of the formation as well as the maintenance of the NMJ, likely to occur owing to misglycosylation of multiple proteins downstream of GFPT1.

Having established a muscle-specific GFPT1 deficient mouse model, we investigated muscle strength and fatigue, and the molecular biochemical effects of GFPT1 deficiency. *Gfpt1^tm1d/tm1d^* mice are not readily distinguishable from control mice with regard to physical appearance, general behaviour and motility, but show a pronounced deficit in muscle strength and a greater susceptibility to fatigue, similar to other mouse models of congenital myasthenia (20–22) and characteristic of human CMS (4,7–9). Whilst patients often present with symptoms at later stages of the disease, *Gfpt1^tm1d/tm1d^* mice present with muscle weakness as early as 6 weeks old. This deficit in mutant mice does not appear to be progressive over the first 6 months, but may progress over longer periods of time. Fatigue induced in the TA muscle following a high number of stimulations together with a pronounced deficit in force produced by the diaphragm muscle from *Gfpt1^tm1d/tm1d^* mice, is indicative of a myopathy, and consistent with EMG recordings in patients with mutations in *GFPT1* as well as other genes in the glycosylation pathway including *ALG2, ALG14, DPAGT1* and *GMPPB* (1,2,4–9,12,23).

We also detected muscle fibre atrophy seen by a progressive replacement of muscle tissue by fibroadipose tissue in the diaphragm muscle from 3 months old mice, which was absent in younger (6 weeks old) mice (data not shown). These findings correspond to the pathological changes observed in muscle biopsies from *DPAGT1*-CMS patients (6). Whilst minor myopathic changes are sometimes seen in some subtypes of CMS, secondary to neurotransmission failure (24,25), more recently, patient muscle MRI studies revealed pronounced myopathic changes in patients with mutations in *GFPT1* and *DPAGT1*, with dystrophic changes reflected by fatty infiltration of the muscle in patients with *GMPPB* mutations (26).

*Gfpt1^tm1d/tm1d^* mice demonstrate changes in endplate architecture illustrated by the fragmentation and reduction in size of AChR clusters in all muscles examined, highlighting the importance of GFPT1 in NMJ differentiation. These pathological changes are observed in a range of muscles regardless of fibre type composition. The explicit morphological changes suggest a synaptopathy that is likely to contribute to the muscle weakness observed in *Gfpt1^tm1d/tm1d^* mice and may become more obvious with disease progression. Notably, fragmentation and smaller endplates are not necessarily indicators of impaired neurotransmission as seen by recent investigations demonstrating that neurotransmission remains unaffected in age-related fragmentation of AChR clusters (27). To investigate the underlying mechanisms that contribute to the observed morphological alterations and subsequently the pathological phenotype observed in *Gfpt1^tm1d/tm1d^* mice, we examined the stability of AChRs. Under normal conditions, AChRs cluster and stabilize on the muscle membrane. Thereafter they are endocytosed, recycled back to the membrane or are degraded (28). Here, we examined whether deficiency of GFPT1 in the *Gfpt1^tm1d/tm1d^* mouse muscle compromises the AChR turnover rate. Whilst there were no significant changes in AChR turnover rate between control and mutant mice, the presence of smaller and fragmented AChRs suggests that trafficking of AChRs to and from the endplate demonstrates some degree of impairment, which is likely to be more prominent if the muscle is exposed to stress.

Additional investigations revealed pathological changes in intercostal muscle NMJs at the ultrastructural level. We observed fewer and simplified junctional folds, a common feature in GFPT1 patients (2,12) and other mouse models of congenital myasthenia (20–22). Since a high density of Na^+^ channels are found in the troughs of the junctional folds, it is possible that simplification of the junctional folds may impair membrane depolarization and thus raise the threshold for muscle action potential generation. Additional pathological changes we observe in *Gfpt1^tm1d/tm1d^* muscle include abnormal variations in fibre size, few regenerating and necrotic fibres, and the presence of tubular aggregates. Our model closely resembles the myopathic phenotype that we see in a patient harbouring the c.686–2A>G mutation which disrupts the longer muscle-specific isoform of GFPT1 resulting in the absence of glycosylated protein expression (2). Subsarcolemmal tubular aggregates are classified as densely packed vesicular or tubular membranes derived from the sarcoplasmic reticulum (29). Not only are they present in muscle biopsies from patients with mutations in proteins involved in the glycosylation pathway (1–3,5,8,9) but are also implicated in myopathies resulting from *STIM1* and *ORAI1* mutations (30–32), and are present in muscle from *Caveolin1*^−/−^ and *Caveolin2*^−/−^ mice (29). Whether tubular aggregates are direct pathological components contributing to the observed phenotype, or whether they represent a compensatory mechanism to pathological events, or a bystander event that is not connected to the functional impairment is poorly understood, making it challenging to understand the molecular mechanisms that induce tubular aggregates (33). In the context of defective glycosylation induced-CMS, one hypothesis is that the tubular aggregates originate from an accumulation of defective proteins consequent to impaired glycosylation. Although we observe a mild predominance of tubular aggregates in Type 2 fibres from *Gfpt1^tm1d/tm1d^*soleus muscles, this observation may become more robust with disease progression. Interestingly, we also discovered the presence of moderately proliferated sarcoplasmic reticulum and Golgi structures in *Gfpt1^tm1d/tm1d^* mouse intercostal muscles. Owing to the subcellular localization of GFPT1 and its role in post-translational protein modifications that take place within these two compartments under normal conditions, aberrant protein accumulation may arise in the cytoplasm of GFPT1 deficient mouse muscle. Similar ectopic proteins suggesting an ER-Golgi-stress response are also observed in caveolin-3-related muscle diseases (34), (G. Coraspe, manuscript in preparation).

The presynaptic alterations we observe include disorganized axons, thinner irregular myelin sheaths and remodelling of motor nerve terminals. Since our model conserves GFPT1 expression in non-muscle tissues, we speculate that the presynaptic changes we observe in *Gfpt1^tm1d/tm1d^* NMJs are secondary to the pathological changes in the postsynaptic apparatus. Previous studies have reported presynaptic alterations owing to a deficiency of muscle derived NMJ proteins. The inducible LRP4 muscle-specific knockout mouse model displays postsynaptic remodelling that is accompanied by presynaptic changes (35,36). Similarly, the muscle-specific conditional β-catenin knockout mouse model demonstrates morphological and functional defects in NMJ nerve terminals, yet there are no presynaptic pathological changes in the motor neuron-specific β-catenin knockout mouse model (37). It is possible that the presynaptic changes we observe in *Gfpt1^tm1d/tm1d^* mice occur owing to defective muscle derived proteins, which could be GFPT1 itself, or misglycosylation of binding partners and activators required for neuronal differentiation and maintaining the integrity of myelin sheaths.

Since glycosylation is a ubiquitous post-translational modification, it is highly probable that GFPT1 is responsible for the glycosylation of proteins other than those required for the formation and maintenance of the NMJ complex. Proteomic profiling of GFPT1 deficient intercostal muscles revealed altered abundance of 2.8% of the quantified proteins affecting different pathways/cellular functions including the ER/SR-Golgi function and protein glycosylation. Here, we identified an upregulation of *O*-GlcNAc transferase (OGT), which is responsible for catalysing the addition of a single GlcNAc to a serine or threonine residue in the O-GlcNAcylation pathway (38), and is regulated by the cellular levels of UDP and UDP-GlcNAc. Previous observations of a distinct decrease of O-GlcNAc proteins are also seen in human GFPT1-deficiency (10). It is likely that the increased expression of OGT in GFPT1 deficient muscle serves as a compensatory mechanism owing to a lack of the precursor donor UDP-GlcNAc. Moreover, our proteomics analyses revealed an increase of glypican-1, a heparan sulphate proteoglycan predominantly expressed in neural tissues and skeletal muscle, and is important for modulating growth factors and influencing axon guidance, Schwann cell myelination and skeletal muscle differentiation (39–41). The increase in glypican-1 in *Gfpt1^tm1d/tm1d^* mouse muscle may serve as a mechanism for skeletal muscle to counter high stress levels, as glypican-1 was previously shown to be upregulated after severe denervation in skeletal muscles as an anti-fibrotic response (42). Interestingly, glypican-1 co-localizes with caveolin-positive endosomal structures displaying a pronounced association upon the initiation of ER-Golgi-stress (43). Notably, via electron microscopic studies, we identified an increase of subsarcolemmal vesicular structures in *Gfpt1^tm1d/tm1d^* mouse muscle that may correspond to caveolae that have not fused with the sarcolemma. This prompted the investigation of the abundance and distribution of subcellular caveolin-3 in GFPT1-deficient muscle showing that the size of the sarcoplasmic puncta immunoreactive for caveolin-3 are significantly larger in mutant muscles compared with controls. As caveolins are required for caveolae-formation (44,45), the larger sarcoplasmic caveolin-3 deposits may influence proper formation of caveolae. Furthermore, tubular aggregates are also present in muscle from *Caveolin1*^−/−^ and *Caveolin2*^−/−^ mice (29), suggesting that caveolin proteins may play a role in preventing the formation of tubular aggregates.

We also see an increase of FNTA, an essential subunit of the geranylgeranyltransferase complex that is implicated in synapse formation by playing an active role in the agrin/MuSK pathway (46). Notably, we see an increase in the expression of MuSK, a protein essential for AChR clustering (21,22,47). The abnormal proteomic profile of GFPT1-deficient mouse muscle allows first insights into dysregulated molecular pathways, but further work is required to elucidate the cellular and subcellular origin of the dysregulated proteins and to elucidate the underlying pathomechanism downstream of GFPT1. Our unbiased proteomic study is limited, as membrane-bound and low-abundant proteins such as the subunits of the AChR may be underrepresented or undetectable. Additional experiments, e.g. glycoproteomics on sub-fractions of enriched membranes may be required to refine the putative glycosylation targets affected by GFPT1 deficiency.

In conclusion, we report a novel GFPT1 deficient mouse model that closely resembles the human *GFPT1*-CMS phenotype on a histological and functional level. Our model provides new insights into proteins that are regulated owing to a complete loss of GFPT1 in muscle, highlights the importance of protein glycosylation in maintaining the integrity of the NMJ and muscle, and provides an enhanced understanding of the pathological and possible compensatory mechanisms occurring in the absence of GFPT1. Importantly, we identify a myopathic phenotype in the *Gfpt1^tm1d/tm1d^* mouse model corresponding to that seen in patients with CMS who harbour mutations in proteins involved in the *N*-glycosylation pathway. Whilst current therapies symptomatically target the myasthenic phenotype, it is unlikely that the myopathy can be reversed. Since GFPT1 is the rate limiting enzyme in the *N*- and *O*-glycosylation pathways, it is plausible that the discoveries made from this model may also be true for other glycosylation defective CMS.

## Materials and Methods

### Mice

All procedures were approved by the Home Office and were carried out in accordance to the Animals Scientific Procedures Act of 1986 under project licence 70/8538. AChR turnover experiments were approved by German authorities and were conducted in accordance with national law (TierSchG7). Mice used for this study were bred in the animal facility at the Functional Genomics Unit, Institute of Genetic Medicine, Newcastle University. Breeders were housed as pairs of one male and one female, or trios of one male and two females. Offspring were housed together in groups of 2–6. *Gfpt1^tm1a^*^(EUCOMM)Wtsi^ and [B6.FVB(129S4)-Tg(Ckmm-cre)5Khn/J] strains were obtained from The Jackson Laboratory. *Gfpt1^tm1c^*^(EUCOMM)Wtsi^ and *Gfpt1^tm1d^*^(EUCOMM)Wtsi^ strains were generated in-house.

### Generation of *Gfpt1^tm1d^*^/^^*tm1d*^ mutant mice and genotyping

All mice used in this study were bred on a C57BL/6N background. The *Gfpt1^tm1c^* allele harbouring a floxed *lacZ-neomycin* trapping cassette was established as part of the International Mouse Phenotyping Consortium, MRC Harwell. This line was crossed with a Cre deleter mouse whereby the *Cre* transgene is driven by the muscle creatine kinase (*Ckm*) promoter, resulting in excision of the floxed exon. The following primers were used for PCR verification of tissue specific Cre-mediated recombination: Control PCR, (F1) 5′ CATGCGTGAACCTGTGTACA 3′ and (R1) 5′ GTCAGAGTTTGCTCACATCA 3′. Mutant PCR, (F1) 5′ CATGCGTGAACCTGTGTACA 3′ and (R2) 5′ GGGTTTCGTAATTGGAAGAG 3′. Control tissues amplified sequences of ∼500 bp, and the mutant amplification was ∼290 bp long. The presence of *Cre* was detected using the following primers: (CreF) 5′ TAAGTCTGAACCCGGTCTGC 3′ and (CreR) 5′ GTGAAACAGCATTGCTGTCACTT 3′, producing a sequence of ∼450 bp. Male mice were used for all experiments in this study.

### Inverted screen measurement

Animals were suspended from an inverted wire grid and the latency to release their grip was recorded. Mice were permitted to hold on for a maximum time of 10 min. Mice that released their grasp before reaching 10 min were given a rest period of 5 min and were given up to two more tries. The maximum hang time was used for further analysis. Data were collected from mice as early as 6 weeks old and animals were tested once a week over a period of 6 months.

### *Ex vivo* isometric tension analysis

Three months old mice were sacrificed via cervical dislocation and a strip of diaphragm muscle from the central tendon to the ribs was excised immediately and assembled in a tissue organ bath (Aurora Scientific) filled with oxygenated Krebs-Ringer solution (154 mM NaCl, 5 mM KCl, 2 mM CaCl_2_, 1 mM MgCl_2_, 11 mM glucose and 5 mM HEPES) at pH 7.4, maintained at 22°C. One end of the diaphragm was attached to a 300C dual-mode servomotor transducer (Aurora Scientific) and the central tendon secured to a rigid post using 4-0 surgical thread. Diaphragm muscles were stimulated by supramaximal 0.2 ms square wave pulses delivered to the nerve via platinum electrodes positioned on either side of the muscle. Data acquisition and control of the servomotor was conducted using a LabView-based DMC program (Dynamic muscle control and Data Acquisition; Aurora Scientific, Version 3.2). We established a force−frequency relationship and assessed muscle resistance to fatigue. The muscle’s optimum length (*L*_o_) was determined using single 0.5 ms isometric twitch stimulations. The maximum isometric tetanic force was established by stimulating the muscle with 0.5 ms pulses at *L*_o_ for a period of 500 ms at 150 Hz. The fatigue protocol involved 100 isometric contractions at a frequency of 150 Hz. This procedure was carried out in accordance with TREAT-NMD protocols.

### *In situ* force measurement

Mice were anaesthetized with an intraperitoneal injection of Hypnorm^®^/Hypnovel^®^/water (1:1:2) at a dosage of 6 µl/g. Anaesthesia was maintained by mask inhalation of isoflurane vaporized at concentrations of up to 4% during surgical procedures and at 0.8–1.3% throughout the rest of the procedure. The distal tendon of the TA muscle was exposed and freed from surrounding fascia and tied with 4-0 surgical braided silk. The sciatic nerve was exposed and all branches were severed except for the common peroneal nerve (CPN) that innervates the TA. The mouse was placed on a heated stage (Aurora Scientific) to maintain body temperature at 37°C. The TA tendon was attached to the lever arm of a 300C dual-mode servomotor transducer (Aurora Scientific). Contractions of the TA muscle were simulated via supramaximal square-wave pulses of 0.2 ms (701B Stimulator; Aurora Scientific) to the distal part of the CPN via bipolar platinum electrodes. Data acquisition and control of the servomotor was conducted using a LabView-based DMC program (Dynamic muscle control and Data Acquisition; Aurora Scientific, Version 3.2).

Muscles were stimulated using a warm up protocol that consisted of 5 stimulations at 50 Hz with a minute rest period between each stimulation. The muscle was subject to a series of single twitches at increasing tensions. The muscle’s optimum length (*L*_o_) was determined and the resting tension that produced the strongest twitch was used throughout the experiment. The force frequency relationship was determined using a series of stimulations at 10, 30, 40, 50, 80, 100, 120, 150 and 180 Hz, each 1 min apart. A stimulation frequency of 120 Hz produced a fully fused tetanus with no reduction in force over the stimulation period. This is the maximum isometric tetanic force (*P*_o_).

The force readings at each frequency recorded in grams (absolute force) were converted to specific force (kN/m^2^). Specific force is the absolute force normalized to CSA of the muscle. The following formula was used to determine the CSA of the muscle: CSA =muscle weight (g)/[optimum TA fibre length (Lf, cm) ×1.06 (g/cm^3^)]. Specific force (N/cm^2^) was calculated using the following formula: specific force (N/cm^2^) =Absolute force (N)/CSA (cm^2^); 1.06 g/cm^3^ is the density of mammalian skeletal muscle. Lf =optimal length (*L*_o_) ×0.6 that represents the fibre length: muscle length ratio for the TA (48).

We also established the force–frequency relationship and assessed the susceptibility of the TA muscle to fatigue. The fatigue protocol involved stimulating the muscle every 2 s at 120 Hz (*P*_o_) 100 times. Force produced upon muscle contraction was recorded after every 10 stimulations. These data are expressed as a percentage of baseline values (force produced after every 10 stimulations). At the end of the experiment, mice were sacrificed via cervical dislocation and muscles were excised and weighed.

### Antibodies

The following antibodies were used in this study.

*Whole-mount muscle staining*: mouse monoclonal anti-neurofilament (1:200, Abcam), rabbit polyclonal anti-synaptophysin (1:100, Thermo Fisher Scientific), Alexa Fluor^®^ 594 α-bungarotoxin conjugate (1:500, Thermo Fisher Scientific), Alexa Fluor^®^ 488 goat anti-mouse (1:500, Thermo Fisher Scientific), Alexa Fluor^®^ 488 goat anti-rabbit (1:500, Life Technologies); *western blotting*: rabbit polyclonal anti-GFPT1 (1:500, Proteintech), mouse monoclonal anti-GAPDH (1:1000, Abcam), rabbit polyclonal anti-glypican 1 (1:1000, Abcam), rabbit polyclonal anti-MuSK (1:1000, Abcam), mouse monoclonal anti-α actinin (1:250, Sigma). Secondary antibodies: IRDye^®^ 800CW Donkey anti-Mouse IgG, IRDye^®^ 680CW goat anti-rabbit IgG; *AChR turnover experiments*: Alexa Fluor^®^ 647 α-bungarotoxin conjugate (25 pmol, Thermo Fisher Scientific) and Alexa Fluor^®^ 488 α-bungarotoxin conjugate (25 pmol, Thermo Fisher Scientific); *Caveolin-3 labelling:* rabbit polyclonal anti-caveolin-3 (1:100, Abcam). Secondary antibodies: Alexa Fluor^®^ 594 goat anti-rabbit (1:100, Thermo Fisher Scientific); *Fibre type identification:* rabbit polyclonal IgG anti-laminin (Sigma 1:750), MHCI (BA-F8 Mouse monoclonal IgG2b) 1:25, MHCIIa (Sc71, Mouse monoclonal IgG1) 1:200, MHCIIx (6H1 Mouse monoclonal IgM) 1:25 and MHCIIb (BF-F3 Mouse monoclonal IgM) 1:200 all from Developmental Studies Hybridoma Bank. Secondary antibodies: Alexa Fluor^®^ 488 IgG goat anti-rabbit (1:500, Thermo Fisher Scientific), Alexa Fluor^®^ 350 IgG2b goat anti-mouse (1:500, Thermo Fisher Scientific), Alexa Fluor^®^ 594 IgG1 goat anti-mouse (1:100, Thermo Fisher Scientific) and Alexa Fluor^®^ 488 IgM goat anti-mouse (1:500, Thermo Fisher Scientific).

### Immunohistochemistry

#### Histology of muscle

Transverse 10 µm sections of TA, intercostal, soleus and EDL muscles were cut using a cryostat (Microm HM 560, Zeiss). Sections were stained with haematoxylin and eosin according to standard procedures. Slides were mounted with DPX Mounting Medium (LAMB) and images were acquired using a Zeiss Axioplan microscope and AxioVision software.

#### Fibre type identification

Transverse 10 µm sections of soleus and TA muscles were cut using a cryostat (Microm HM 560, Zeis) and labelled for Myosin Heavy Chains (MHC) MHCI, MHCIIa, and MHCIIb and MHCIIx (49). Sections were blocked (10% normal goat serum in PBS) for 1 h at room temperature and then incubated with the following primary antibodies: anti-laminin, anti-MHCI, anti-MHCIIa, anti-MHCIIx and anti-MHCIIb for 1 h at room temperature. Sections were washed in PBS and incubated in secondary antibodies for 1 h. Sections were then washed and mounted using Vectashield hardset mounting medium (Vector Laboratories). Tiled images of the whole sections were captured using a Zeiss Axio Imager fluorescent microscope with Zen software and analysed using ImageJ analysis software.

#### Whole-mount muscle staining

Whole muscles were dissected and fixed (1% paraformaldehyde in 0.1 M phosphate buffer) for 30 min. They were then incubated with Alexa Fluor^®^ 594 α-bungarotoxin conjugate in Liley’s solution (12 mM NaHCO_3_, 4 mM KCl, 1 mM KH_2_PO_4_, 138.8 mM NaCl, 1 mM MgCl_2_, 2 mM CaCl_2_, 11 mM glucose; Sigma-Aldrich) for 1 h. Muscle fibres were teased into small bundles during washes. Muscles were permeabilized in ethanol followed by methanol (10 min at −20°C), followed by an incubation in 0.1% Triton X-100 in PBS (for 15 min, room temperature). Muscles were incubated overnight with antibodies against neurofilament and synaptophysin in PBS containing 3% BSA (Sigma-Aldrich) and 0.1 M lysine (Sigma-Aldrich). The next day muscles were incubated in Alexa Fluor^®^ 488 secondary antibodies for 3 h at room temperature and washed in PBS overnight. The muscles were mounted on slides with Vectashield mounting medium with DAPI (Vector Laboratories). Samples were visualized using a Nikon A1R laser scanning confocal microscope. Laser power and parameter settings were kept constant and Z-stack images were acquired and processed using NIS-elements AR 4.20.02 software. For quantification of the AChR cluster area, single-projected images derived from overlaying image stacks were analysed by ImageJ analysis software using a thresholding approach.

#### Caveolin-3 labelling

Cryosections from the soleus and TA muscles were taken at 10 μm using a CM1860 cryostat (Leica) and mounted on Histobond adhesion microscope slides. Sections were permeabilized (0.5% Triton X-100 in TBS) for 10 min and blocked (10% normal goat serum in TBS) for 30 min at room temperature, and then incubated with an antibody against caveolin-3 for 2 h at room temperature. Sections were washed and incubated in secondary antibodies for 1 h at room temperature. Sections were then washed and mounted using Vectashield hardset mounting medium (Vector Laboratories). Tiled images were acquired using a Zeiss Axioplan microscope and camera. Analysis was performed using ImageJ and GraphPad Prism v7. The number of fibres expressing caveolin-3 was counted and determined relative to the total number of fibres present. The size of caveolin-3 punctate was determined using a thresholding approach.

### Transmission electron microscopy

Fresh tissue samples of intercostal muscles were fixed in 3.9% buffered glutaraldehyde, osmicated in 1% phosphate-buffered osmium tetroxide, dehydrated and embedded in epoxy resin. One micrometre of semi-thin sections was stained with toluidine blue. Ultrathin sections (100 nm) of at least one transverse and one longitudinal block per animal were contrasted by uranyl acetate and lead citrate as previously described (50). Electron microscopy images were recorded using a CM10 transmission electron microscopy (Philips, Amsterdam, the Netherlands).

### *In vivo* visualization and measurement of AChR turnover rate

Mice were anaesthetized under isoflurane vaporized at concentrations of 0.6–1.5%, and administered an intramuscular injection of 25 pmol of α-bungarotoxin 488 (green) in the TA muscle. Ten days later, mice were administered a combination of Xylazin (Bayer) and Zoletil (Laboratories Virba) by intraperitoneal injection, and administered an intramuscular injection of 25 pmol α-bungarotoxin 647 (red). One hour following the second administration, superficial TA muscles were examined using microscopy. Briefly, 3D stacks at 512 × 512 pixel resolution were taken of α-bungarotoxin 488 signals (old receptors) and of α-bungarotoxin 647 signals (new receptors) using a 63× objective and confocal *in vivo* imaging. The 3D stacks were automatically segmented using a custom-made algorithm and pixel signal intensity values for each channel were extracted. The fraction of pixels per NMJ was calculated.

### Western blots

Tissues were homogenized in lysis buffer (RIPA buffer, ThermoScientific) and protease inhibitors (Complete EDTA-free protease inhibitor, Roche) using a tissue raptor (Qiagen). Samples were centrifuged and the protein-containing supernatant was collected for immunoblot analyses. Proteins were resolved by SDS-PAGE on Novex NuPAGE 4–12% Bis–Tris Gels (Thermo Fisher Scientific) for 45 min at 200 V, and transferred onto PVDF membranes (Licor). Membranes were blocked with TBS blocking buffer (Licor) and incubated with primary antibodies in blocking solution containing 0.2% Tween-20, overnight at 4°C. After washing with TBS/Tween-20, membranes were incubated with IRDye^®^ 800CW Donkey anti-Mouse IgG, IRDye^®^ 680CW Goat anti-Rabbit IgG secondary antibodies diluted in TBS blocking buffer containing 0.2% Tween-20 and 0.01% SDS for 1 h. Membranes were washed with TBS/Tween-20 and TBS, and immunoreactive proteins were detected using an Odyssey infrared scanner (Licor Biosciences).

### Proteomic profiling of GFPT1 deficient intercostal muscles

Proteomic profiling was conducted using a label-free LC-MS/MS. Materials and methods are described in Supplementary Material, Resource S14.

### Statistical analysis

Statistical analyses were performed with IBM SPSS Statistics 22.0 software. Data were analysed using a two-sample *t*-test when variances did not differ between samples. An independent sample Mann–Whitney *U* test was performed to compare myofibre size variation as the data did not follow a normal distribution. Proteomic data were analysed as described in Supplementary Material, Resource S1. *P* < 0.05 was considered statistically significant.

## Supplementary Material

Supplementary Data
