# Supplementary material for: GFPT1 deficiency in muscle leads to myasthenia and myopathy in mice
Source: Hum Mol Genet. 2018 Jun 14;27(18):3218–32. doi: 10.1093/hmg/ddy225 (PMC6121184; doi:10.1093/hmg/ddy225)
Supplement: Supplementary Data [file ddy225_supp.zip › EMS_1.pdf]

GFPT1 deficiency in muscle leads to myasthenia and myopathy in mice

Human Molecular Genetics

Yasmin Issop[1], Denisa Hathazi[2], Muzamil Khan[3], Rüdiger Rudolf [3,4,5], Joachim Weis[6], Sally Spendiff[1], Clarke R. Slater[7], Andreas Roos[1,2], Hanns Lochmüller[1,8,9]

Affiliations: 1 - John Walton Muscular Dystrophy Research Centre, MRC Centre for Neuromuscular Diseases, Institute of Genetic Medicine, Newcastle University, Newcastle upon Tyne. 2 - Leibniz-Institut für Analytische Wissenschaften—ISAS e.V, Otto-Hahn-Str. 6b, 44227 Dortmund, Germany. 3 - Institute of Toxicology and Genetics, Karlsruhe Institute of Technology, 76344 Eggenstein-Leopoldshafen, Germany. 4 - Interdisciplinary Center for Neurosciences, University of Heidelberg, 69120 Heidelberg, Germany. 5 - Institute of Molecular and Cell Biology, Mannheim University of Applied Sciences, 68163 Mannheim, Germany. 6 - Institute of Neuropathology, RWTH Aachen University Hospital, Pauwelsstr. 30, 52074, Aachen, Germany. 7 - Institute of Neuroscience, Newcastle University, Newcastle upon Tyne. 8- Department of Neuropediatrics and Muscle Disorders, Medical Center – University of Freiburg, Faculty of Medicine, Mathildenstr. 1, 79160 Freiburg, Germany. 9 -Centro Nacional de Análisis Genómico (CNAG-CRG), Center for Genomic Regulation, Barcelona Institute of Science and Technology (BIST), Barcelona, Catalonia, Spain

Corresponding author: hanns.lochmuller@gmail.com

Contents

Online Resource 1: Generation of *Gfpt1*$^{tm1c/tm1c}$ mice

Online Resource 2: Primers used for genotyping

Online Resource 3: Combinations of primers used for genotyping and expected band size

Online Resource 4: Mice harbouring the *Ckm-Cre* transgene do not exhibit a muscle phenotype

Online Resource 5: Force frequency relationship in control and *Gfpt1*$^{tm1d/tm1d}$ mice

Online Resource 6: Muscle fibre type and tubular aggregate predominance

Online Resource 7: Postsynaptic changes in *Gfpt1*$^{tm1d/tm1d}$ mouse muscle accompanied by presynaptic alternations

Online Resource 8: AChR turnover in TA muscles from control and *Gfpt1*$^{tm1d/tm1d}$ mice

Online Resource 9: Regulated proteins with N- and O-glycosylation sites

Online Resource 10: Localisation of regulated proteins in intercostal muscles from *Gfpt1*$^{tm1d/tm1d}$ mice.

Online Resource 11: Schematic representation showing localisation of regulated proteins in intercostal muscles from in *Gfpt1*$^{tm1d/tm1d}$ mice

Online Resource 12: Proteomic profiling of GFPT1 deficient intercostal muscles

Online Resource 13: Schematic representation showing affected pathways in GFPT1 deficient intercostal muscles.

Online Resource 14: Supplementary material

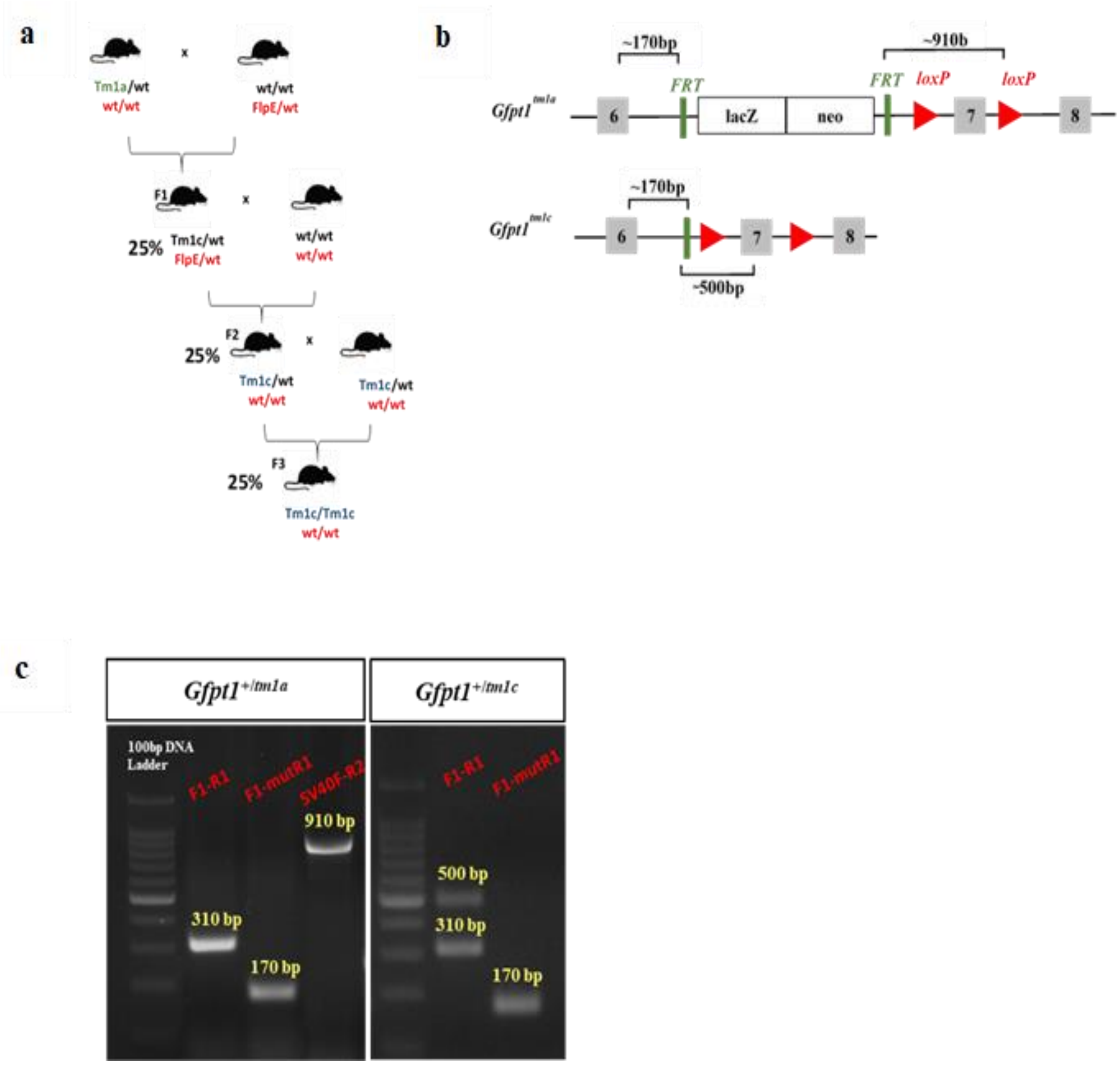

Online Resource 1. Generation of *Gfpt1*$^{tm1c/tm1c}$ mice. (a) *Gfpt1*$^{+/tm1a}$ mice were crossed with mice hemizygous for FlpE recombinase [B6;SJL-Tg(ACT*Flpe*)9205Dym/J] resulting in the conversion of the *Gfpt1*^{tm1a} allele to the *Gfpt1*^{tm1c} allele (F1). *Gfpt1*$^{+/tm1c}$ mice carrying the *Flp* transgene were crossed with wild type mice. *Gfpt1*$^{+/tm1c}$ offspring (F2) free of FlpE recombinase were selected and crossed to generate *Gfpt1*$^{tm1c/tm1c}$ mice (F3). Homozygous *Gfpt1*$^{tm1c/tm1c}$ mice were obtained after 3 generations of breeding. (b) Schematic representation of the targeting vector. Exons are shown in numbered rectangles and the positions of the inserted FRT and loxP sites are shown. The positions of primers used for genotyping and the length of the amplified PCR products in *Gfpt1*^{tm1a} and *Gfpt1*^{tm1c} are indicated. (c) PCR on genomic DNA extracted from ear clips showing recombination events resulting in the conversion of the *Gfpt1*^{tm1a} allele to the *Gfpt1*^{tm1c} allele. PCR products in wildtype (~310bp), *Gfpt1*^{tm1a} (~910bp) and *Gfpt1*^{tm1c} (~500bp) are shown. All transgenic mice produce bands of ~170bp. *Forward primers*: F1, SV40F. *Reverse primers*: R1, mutR1, R2.

Online Resource 2. Primers used for genotyping

Primer name	Primer Sequence (5’-3’)
**F1**	CAT GCG TGA ACC TGT GTA CA
**SV40-F**	CGC GTC GAG AAG TTC CTA TT
**R1**	GTC AGA GTT TGC TCA CAT CA
**mutR1**	GCT TCA AGG ATA AGG CTT CAA G
**R2**	GGG TTT CGT AAT TGG AAG AG
**FlpE-F1**	GGA CCG GCA ATT CTT CAA GCA
**FlpE-R1**	CCA CGG CAG AAG CAC GCT TAT
**CreF**	TAA GTC TGA ACC CGG TCT GC
**CreR**	GTG AAA CAG CAT TGC TGT CAC TT

Online Resource 3. Combination of primers used for genotyping and expected band size

**Genotype**	**Forward Primer**	**Reverse Primer**	**Band Size**
**Wild type**	F1	R1	~310bp
**All targeted alleles**	F1	mutR1	~170bp
Gfpt1^{tm1a}	SV40-F	R2	~910bp
Gfpt1^{tm1c}	F1	R1	~500bp
Cre	CreF	CreR	~450bp
Flp	FlpE-F1	FlpE-R1	~230bp

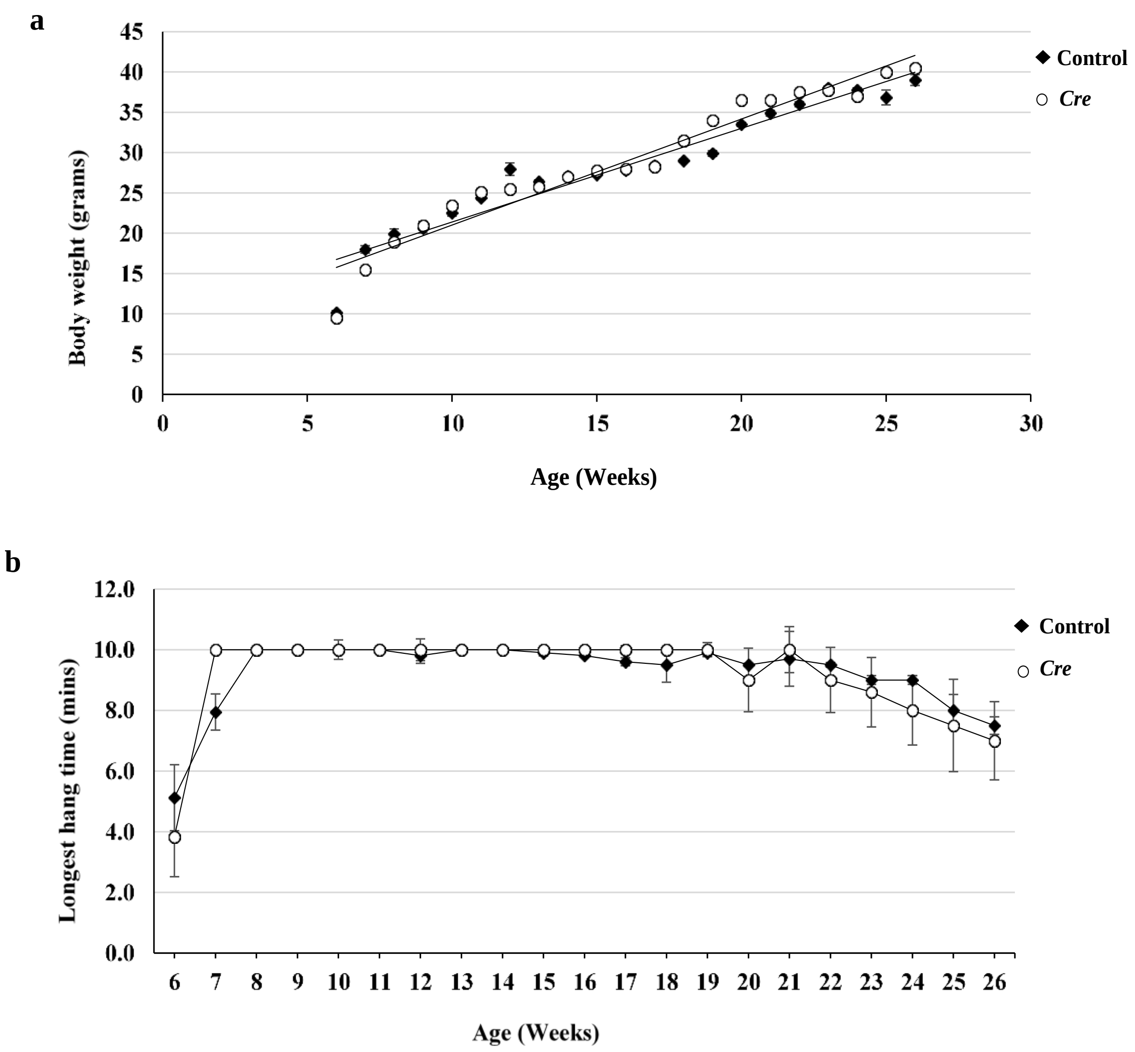

Online Resource 4. Mice harbouring the *Ckm*-Cre transgene do not exhibit a muscle phenotype. (a) Growth curves of control mice (n = 8) and *Ckm*-Cre mice (n= 6). (b) The latency for control mice (n = 8) and *Ckm*-Cre mice (n = 6) to fall from a wire grid at various time points. Data are mean + SEM. $p > 0.05$. ns, not significant at any time point.

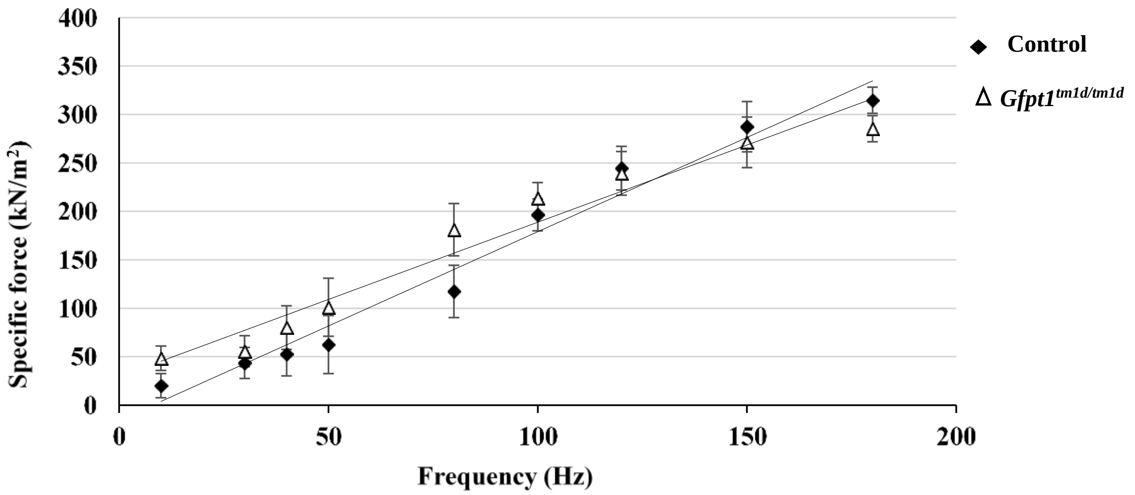

Online Resource 5. Force frequency relationship in control and *Gfpt1*$^{tm1d/tm1d}$ mice. Mean specific force produced by the TA muscle following tetanic stimulation of the CPN at increasing stimulation frequencies of 10, 30, 40, 50, 80, 100, 120, 150 and 180 Hz, with a delay of 1 minute between each stimulation (n = 5). Data are mean + SEM. p > 0.05. ns, not significant at any frequency.

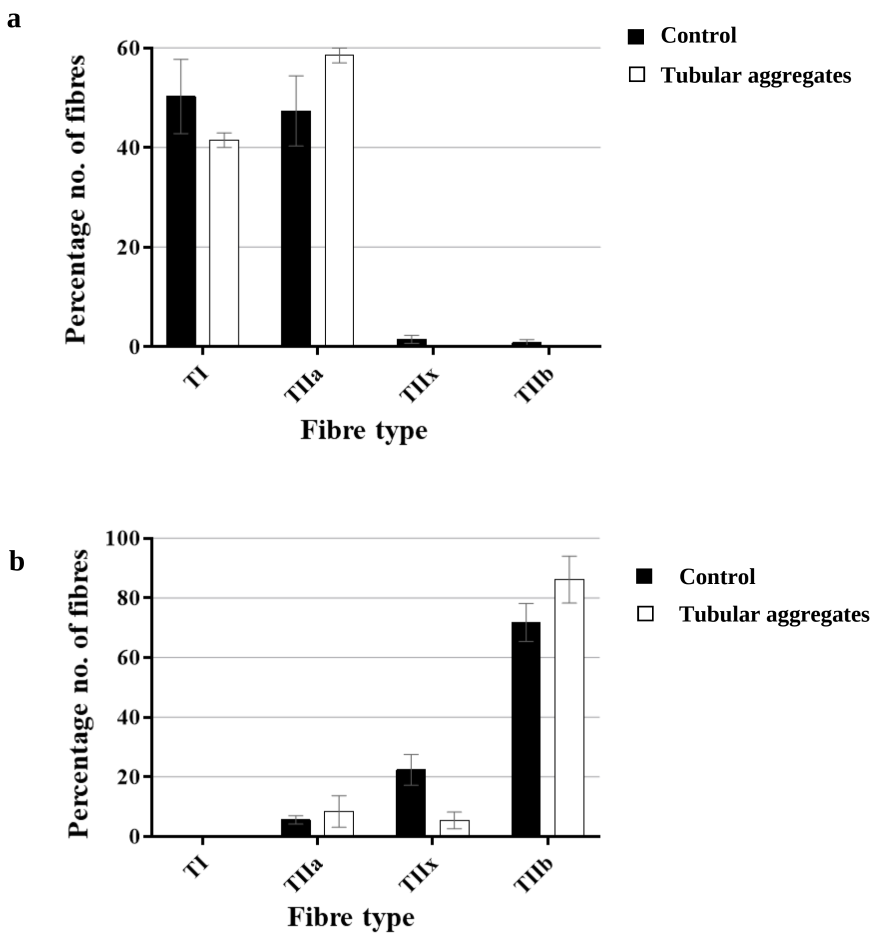

Online Resource 6. Muscle fibre type and tubular aggregate predominance. No significant tubular aggregate fibre type specific predominance was observed in the soleus (n = 4) (a), and TA muscles (n = 4) (b) from *Gfpt1*$^{tmld/tmld}$ mice compared to controls. $p > 0.05$. ns, not significant.

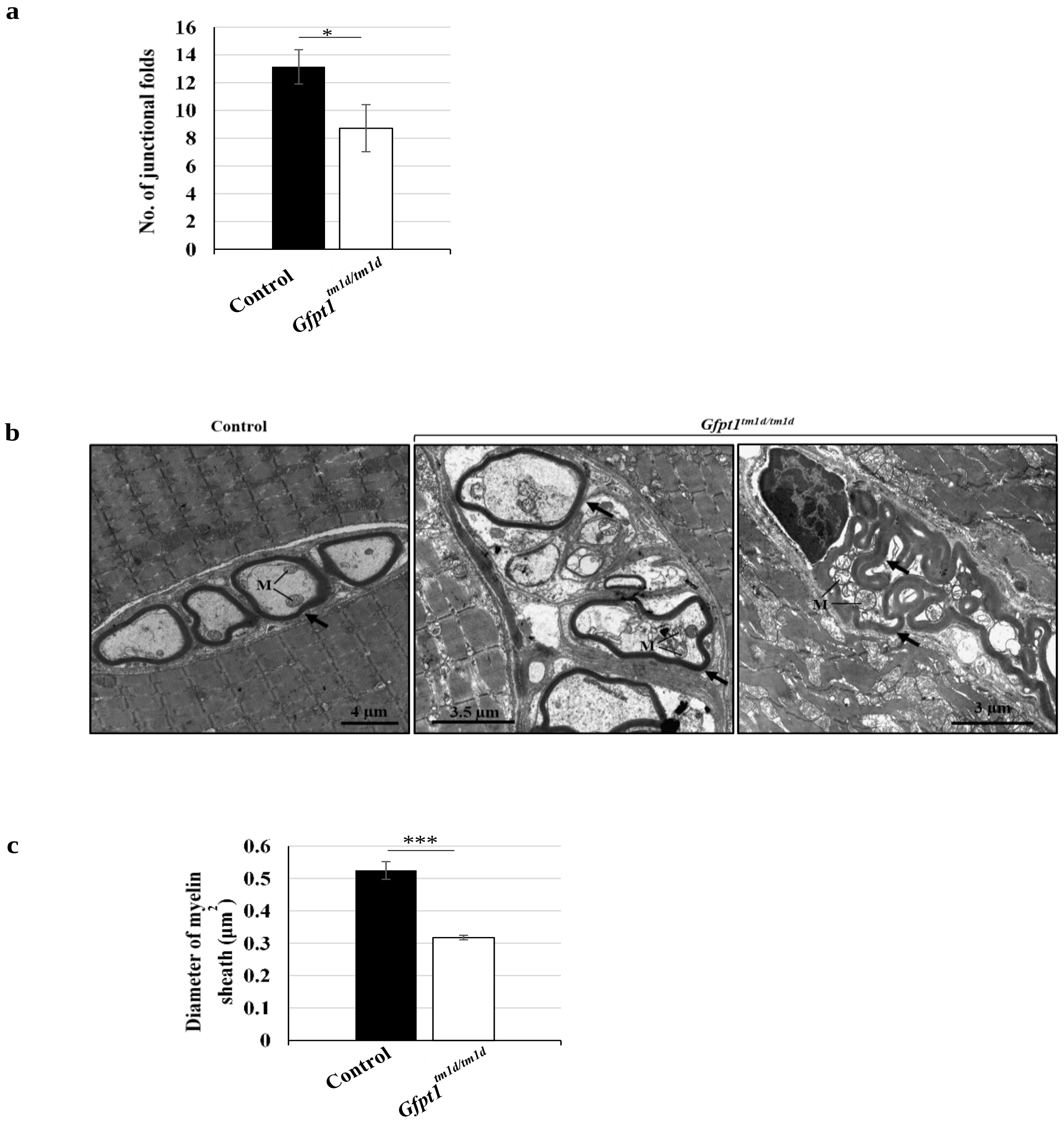

Online Resource 7. Postsynaptic changes in *Gfpt1*$^{tm1d/tm1d}$ mouse intercostal muscles accompanied by presynaptic alternations. (a) Quantitative analysis revealed significantly fewer postsynaptic junctional folds [no. of folds/terminal, control (n = 21); *Gfpt1*$^{tm1d/tm1d}$ (n = 15)], (b) irregular and occasionally convoluted presynaptic myelin sheaths, and (c) a significant reduction in the diameter of myelin sheaths, in *Gfpt1*$^{tm1d/tm1d}$ mouse muscle compared to controls. Mitochondria (M), myelin sheaths (black arrows). Data are mean $\pm$ SEM. $*p < 0.05$, $***p < 0.001$.

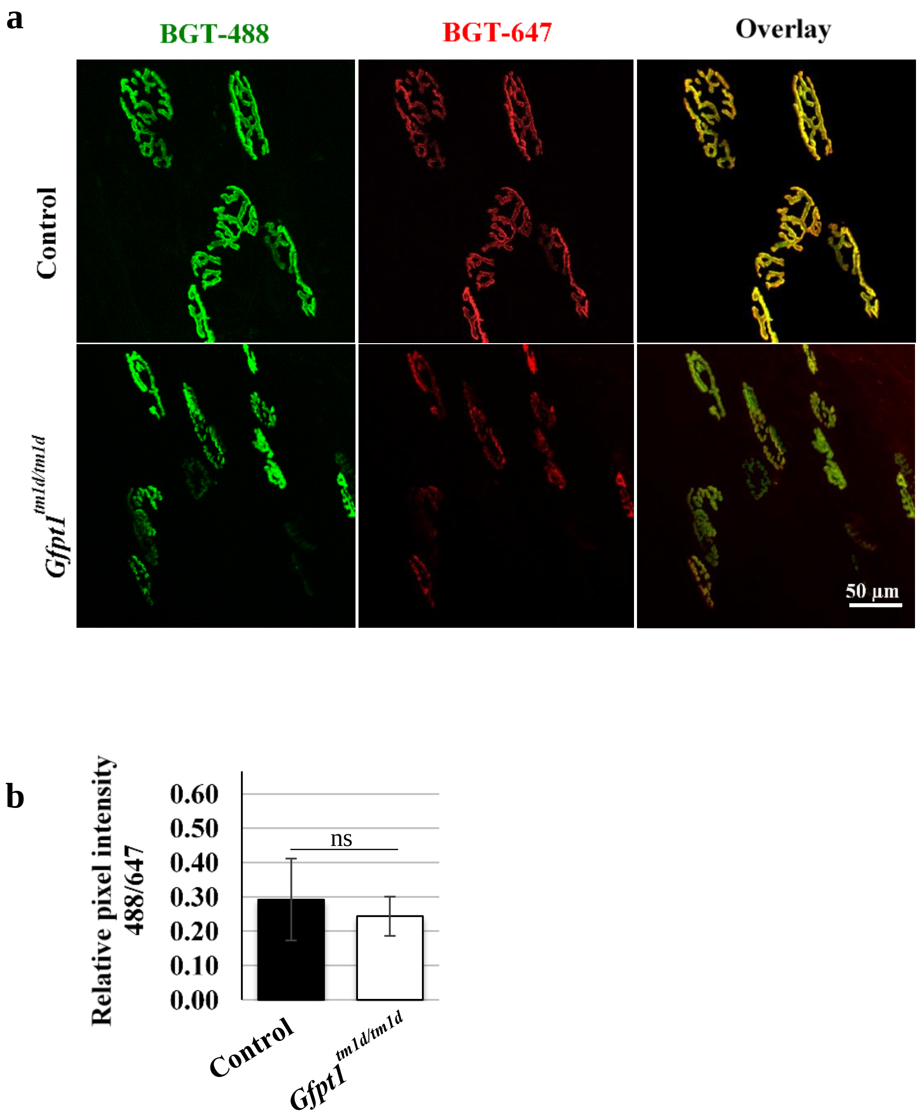

Online Resource 8. AChR turnover in TA muscles from control and *Gfpt1*$^{tm1d/tm1d}$ mice. (a) Representative images of old-receptor signals labelled with BGT-488 (green), new-receptor signals labelled with BGT-647 (red) and overlay. (b) Quantification of relative pixel intensity between control and *Gfpt1*$^{tm1d/tm1d}$ mouse muscle (n = 3). Data are mean $\pm$ SEM. $p > 0.05$, ns, not significant.

Online Resource 9. Regulated proteins with N- and O-glycosylation sites.

Protein	Gene	Abundance	Type and no. of glycosylation sites
Aminopeptidase N	*Anpep*	Upregulated	N- GlcNAc (17)
Complement C4-B	*C4b*	Upregulated	N- GlcNAc (4)
Glypican-1	*Gpc1*	Upregulated	N- GlcNAc (2)<br>O-Xylose (3)
Vesicular integral-membrane protein	*Lman2*	Upregulated	N- GlcNAc (1)
Vitronectin	*Vtn*	Upregulated	N- GlcNAc (3)
UDP-N-acetylglucosamine--peptide N-acetylglucosaminyltransferase	*Ogt*	Upregulated	O- GlcNAc (2)
Uncharacterized family 31 glucosidase KIAA1161	*Kiaa1161*	Downregulated	N- GlcNAc (3)

Online Resource 10. Localisation of regulated proteins in intercostal muscles from *Gfpt1*$^{tm1d/tm1d}$ mice. Proteins 1-29 are upregulated with 2 or more unique peptides, 30-39 are upregulated with 1 unique peptide, 40-43 are downregulated with 1 unique peptide.

	**Protein**	**Subcellular Localisation**
1	**Galectin-3**	Cytoplasm, extracellular space, nucleus
2	**UDP-N-acetylglucosamine-peptide N-acetylglucosaminyltransferase**	Cytoplasm, nucleus, plasma membrane
3	**Plastin-2**	Cytoplasm
4	**Actin-related protein 2/3 complex subunit 1B**	Cytoplasm
5	**Vitronectin**	Extracellular space
6	**Perilipin-1**	ER-Golgi network
7	**Membrane primary amine oxidase**	Plasma membrane
8	**Histone H1.5**	Nucleus
9	**BTB/POZ domain-containing protein KCTD12**	Extracellular space, plasma membrane
10	**Rho GDP-dissociation inhibitor 2**	Cytoplasm
11	**Sec1 family domain-containing protein 1**	Cytoplasm, ER-Golgi network
12	**Annexin A1**	Cytoplasm, nucleus, plasma membrane
13	**Protein farnesyltransferase/geranylgeranyltransferase type-1 subunit alpha**	Cytoplasm, plasma membrane
14	**Macrophage-capping protein**	Cytoplasm, nucleus
15	**Glypican-1**	Plasma membrane
16	**Cysteine-rich protein 1**	Cytoplasm
17	**Ras GTPase-activating-like protein IQGAP1**	Plasma membrane
18	**Vesicular integral-membrane protein VIP36**	ER-Golgi network
19	**Complement C4-B**	Extracellular space
20	**Tubulin-specific chaperone A**	Cytoplasm
21	**Aminopeptidase N**	Plasma membrane
22	**SH3 domain-binding glutamic acid-rich-like protein 3**	Cytoplasm, nucleus
23	**Syntaxin-7**	Plasma membrane
24	**Coatomer subunit gamma-1**	Cytoplasm, ER-Golgi network
25	**Ras-related protein Rab-7a**	Cytoplasm
26	**Myristoylated alanine-rich C-kinase substrate**	Cytoplasm, plasma membrane
27	**Transcription intermediary factor 1-beta**	Nucleus
28	**Coatomer subunit beta**	Cytoplasm, ER-Golgi network, plasma membrane
29	**Protein disulfide-isomerase A3**	ER-Golgi network
30	**60S ribosomal protein L3**	Cytoplasm, nucleus
31	**Periostin**	ER-Golgi network
32	**Complement C1q subcomponent subunit B**	Extracellular space
33	**Coiled-coil domain-containing protein 127**	ER-Golgi network, extracellular space
34	**Bone marrow proteoglycan**	Cytoplasm

	**Protein**	**Subcellular Localisation**
35	**Minor histocompatibility antigen H13**	ER-Golgi network, plasma membrane
36	**KN motif and ankyrin repeat domain-containing protein 2**	Cytoplasm, mitochondria
37	**Heme oxygenase 2**	ER-Golgi network
38	**Histone H1.3**	Nucleus
39	**Cytochrome P450 20A1**	Plasma membrane
40	**Selenoprotein T**	ER-Golgi network
41	**Fructosamine-3-kinase**	Cytoplasm
42	**Uncharacterized family 31 glucosidase KIAA1161**	Plasma membrane
43	**Nitric oxide synthase, brain**	Plasma membrane

Online Resource 11. Schematic representation showing localisation of regulated proteins in intercostal muscles from in *Gfpt1*$^{tm1d/tm1d}$ mice.

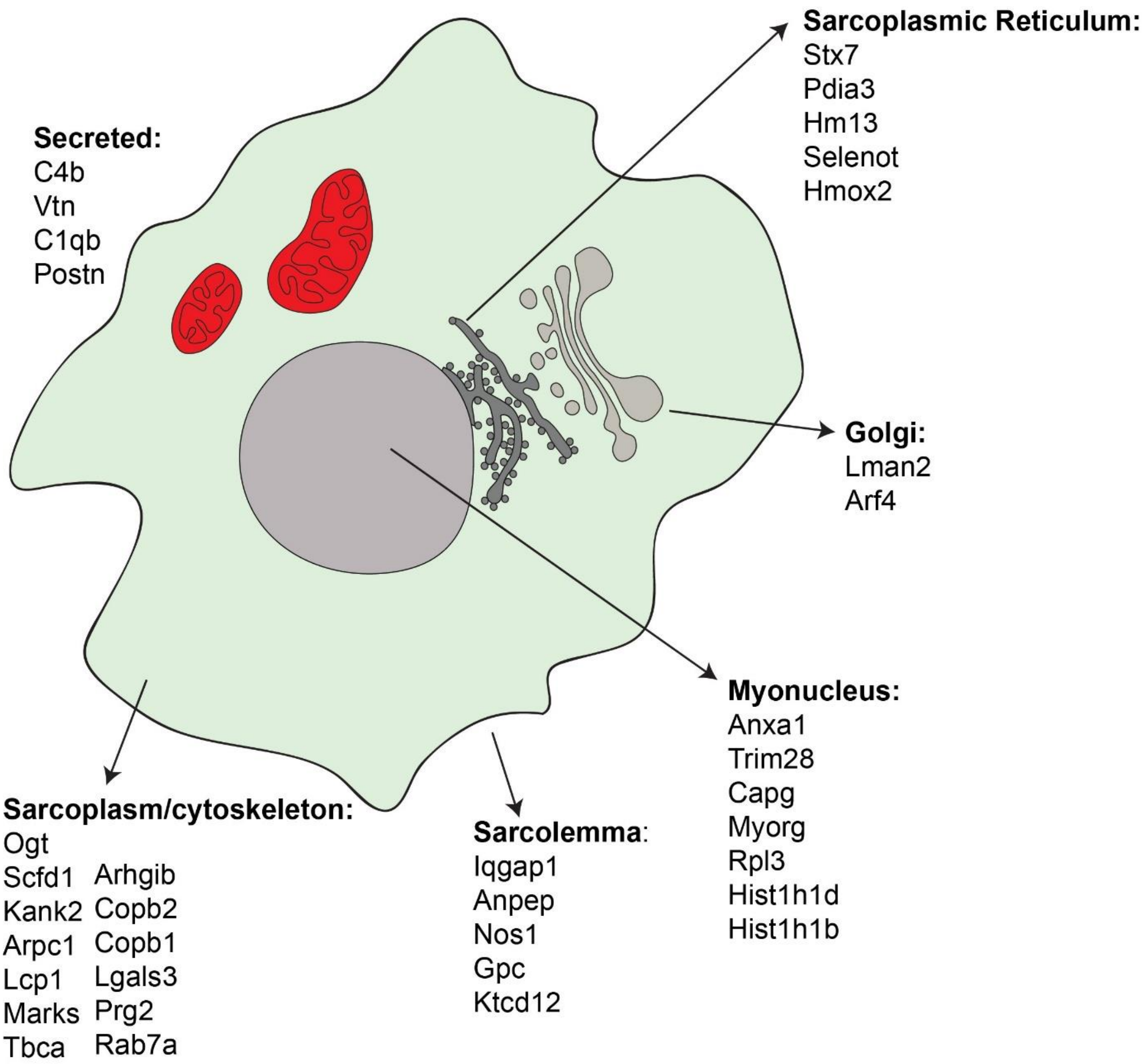

Online Resource 12. Proteomic profiling of GFPT1 deficient intercostal muscles **(**See supplementary Microsoft Excel File).

Online Resource 13. Schematic representation showing affected pathways in GFPT1 deficient intercostal muscles.

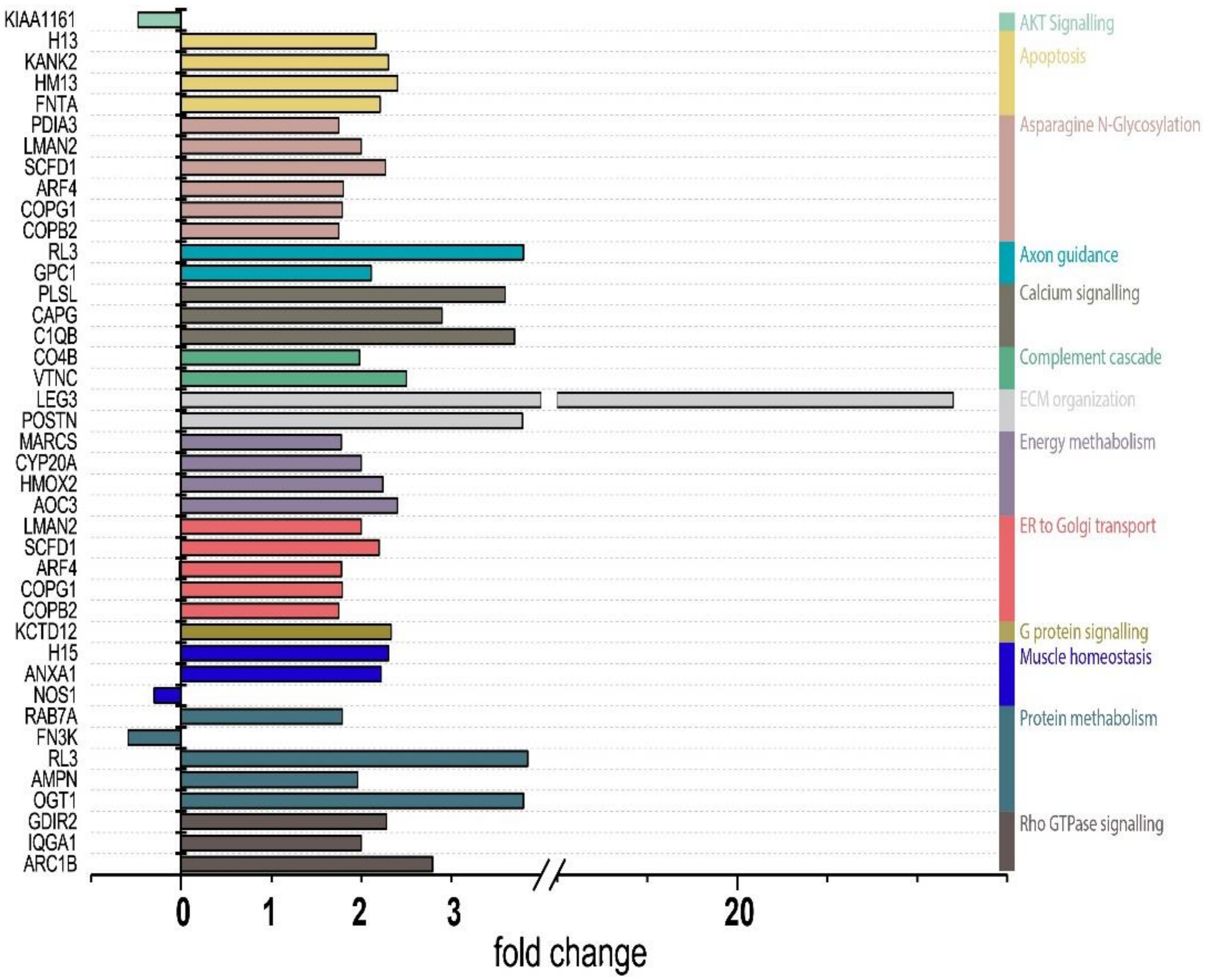

Online Resource 14: Supplementary material

Materials and methods proteomic profiling of GFPT1 deficient intercostal muscles

Reagents

Ammonium hydrogen carbonate (NH_4HCO_3), anhydrous magnesium chloride ($MgCl_2$), guanidine hydrochloride (GuHCl), iodoacetamide (IAA), and urea were purchased from Sigma-Aldrich, Steinheim, Germany. Tris base was obtained from Applichem Biochemica, Darmstadt, Germany and Sodium dodecyl sulfate (SDS) was purchased from Carl Roth, Karlsruhe, Germany. Dithiothreitol (DTT), EDTA-free protease inhibitor (Complete Mini) tablets were obtained from Roche Diagnostics, Mannheim, Germany. Sodium chloride (NaCl) and calcium chloride ($CaCl_2$) were from Merck, Darmstadt. Sequencing grade modified trypsin was from Promega, Madison, WI USA. Benzonase® Nuclease was purchased from Novagen. Bicinchoninic acid assay (BCA) kit was acquired from Thermo Fisher Scientific, Dreieich, Germany. All chemicals for ultra-pure HPLC solvents such as formic acid (FA), trifluoroacetic acid (TFA) and acetonitrile (ACN) were obtained from Biosolve, Valkenswaard, The Netherlands.

Cell lysis, sample preparation and trypsin digestion

In total six samples, *i.e.* three intercostal muscle samples derived from the GFPT1 mouse model and three derived from wildtype littermates were processed independently. Approximately 5 mg of muscle tissue was lysed in 500 µL of a buffer containing 50 mM Tris-HCl (pH 7.8), 150 mM NaCl, 1 % SDS, and Complete Mini using a manual glass grinder. Afterwards, samples were centrifuged for 5 min at 4°C and 5000 *g*. Protein concentration of the supernatant was determined by BCA assay (according to the manufacturer's protocol). Cysteines were reduced by addition of 10 mM DTT and subsequent incubation at 56°C for 30 min. Free thiol groups were alkylated with 30 mM IAA at room temperature (RT) in the dark for 30.

Sample preparation were performed using filter-aided sample preparation (FASP) (Wisniewski *et al.*, 2009) with some minor changes. Briefly, 100 µg of protein lysate was diluted 10-fold with freshly prepared buffer composed of 8 M urea/100 mM Tris-HCl (pH 8.5) (Kollipara and Zahedi, 2013) and placed on a Microcon centrifugal device (30 KDa cutoff). Afterwards, the filter was centrifuged at 13,500 *g* at RT for 15 min (all the following centrifugation steps were performed under the same conditions). Three washing steps were carried out with 100 µL of 8 M urea/100 mM Tris-HCl (pH 8.5). For buffer exchange, the device was washed thrice with 100 µL of 50 mM NH_4HCO_3 (pH 7.8). Digestion buffer (final volume of 100 µL) containing trypsin (Promega) (1:25 w/w, protease to substrate), 0.2 M GuHCl and 2 mM $CaCl_2$ in 50 mM NH_4HCO_3 (pH 7.8) was added to the concentrated proteins and the samples were incubated at 37°C for 14 h. Resulting tryptic peptides were recovered by centrifugation with 50 µL NH_4HCO_3 (50 mM) followed by 50 µL of ultra-pure water. Finally,

acidification of the peptides was achieved by addition of 10 % TFA (v/v). The digests were quality controlled as described previously (Burkhart *et al.*, 2012).

LC-MS/MS analysis

Samples were measured using an Ultimate 3000 nano RSLC system coupled to an Orbitrap Fusion Lumos mass spectrometer (both Thermo Scientific) and analyzed in a randomized order to minimize systematic errors. Briefly, peptides were pre-concentrated on a 100 µm x 2 cm C18 trapping column for 10 min using 0.1 % TFA (v/v) at a flow rate of 20 µL/min followed by separation on a 75 µm x 50 cm C18 main column (both Pepmap, Thermo Scientific) with a 120 min LC gradient ranging from 3-35 % of 84 % ACN, 0.1 % FA (v/v) at a flow rate of 230 nL/min. MS survey scans were acquired in the Orbitrap from 300 to 1500 m/z at a resolution of 120000 using the polysiloxane ion at *m/z* 445.12003 as lock mass (Olsen *et al.*, 2005), an automatic gain control target value of $2.0x10^5$ and maximum injection times of 50 ms. Top speed most intense signals were selected for fragmentation by HCD with a collision energy of 30 % and MS/MS spectra were acquired in the Orbitrap using a target value of $2.0x10^3$ ions, a maximum injection time of 300ms and a dynamic exclusion of 15 s.

Label free data analysis

Data analysis of the acquired label free quantitative MS data was performed using the Progenesis LC-MS software from Nonlinear Dynamics (Newcastle upon Tyne, UK). Alignment of MS raw data was conducted by Progenesis which automatically selected one of the LC-MS files as reference. After peak picking, only features within retention time and *m/z* windows from 0-120 min and 300-1500 m/z, with charge states +2, +3, and +4 were considered for peptide statistics, analysis of variance (ANOVA). MS/MS spectra were exported as peak lists. To maximize the number of identified peptides and proteins at a given quality we used our PeptideShaker software 0.28.0 (http://code.google.com/p/peptide-shaker/) for interpretation of peptide and protein identifications. Therefore, peak lists were searched against a concatenated target/decoy version of the mouse Uniprot database using Mascot 2.4 (Matrix Science), MS-GF+, and X!Tandem Jackhammer (2013.06.15) with the help of searchGUI 1.14.4 (Vaudel*et al.*, 2011). Trypsin with a maximum of two missed cleavages was selected as enzyme. Carbamidomethylation of Cys was set as fixed and oxidation of Met was selected as variable modification. MS and MS/MS tolerances were set to 10 ppm and 0.5 Da, respectively. Combined search results were filtered at a false discovery rate (FDR) of 1 % on the protein level and exported using the advanced PeptideShaker features that allow direct re-import of the quality-controlled data into Progenesis. Peptide sequences containing oxidized Met were excluded for further analysis. Only proteins that were quantified with unique peptides were exported. For each protein in the following step, average of the normalized abundances (obtained from Progenesis) from the analyses was calculated to determine the ratios between the GFPT1 mutant mice and controls. Only proteins which

were (*i*) commonly quantified in all the replicates with (*ii*) one unique peptide, (*iii*) an ANOVA p-value of <0.05 (Progenesis) and (*iv*) an average ratio < 1.75 or > 0.66 were considered as regulated.
